# Sentinel Equines in Anthropogenic Landscapes: Bioaccumulation of Heavy Metals and Hematological Biomarkers as Indicators of Environmental Contamination

**DOI:** 10.3390/toxics13121064

**Published:** 2025-12-09

**Authors:** Maria Popescu, Mirela Alexandra Tripon, Alexandru Florin Lupșan, Denisa Bungărdean, Cristian Mihăiță Crecan, Mihai Musteata, Paula Maria Pașca, Sorin Marian Mârza, Rober Cristian Purdoiu, Ionel Papuc, Radu Lăcătuș, Caroline Maria Lăcătuș, Luciana Cătălina Panait, Teodora Sonia Patrichi, Ileana-Rodica Matei, Cristian-Radu Sisea, Claudiu Ioan Bunea, Anamaria Călugăr, Ioan Valentin Petrescu-Mag, Zsofia Daradics, Florin-Dumitru Bora

**Affiliations:** 1 Equine Clinic, Faculty of Veterinary Medicine, University of Agricultural Sciences and Veterinary Medicine (UASVM) Cluj-Napoca, 3-5 Mănăștur Street, 400372 Cluj-Napoca, Romania; maria.popescu@usamvcluj.ro; 2 Department of Reproduction, Obstetrics and Veterinary Gynecology, Faculty of Veterinary Medicine, University of Agricultural Sciences and Veterinary Medicine (UASVM) Cluj-Napoca, Mănăştur Street 3-5, 400372 Cluj-Napoca, Romania; mirela.tripon@usamvcluj.ro; 3 Department of Anesthesiology and Surgery, University of Agricultural Sciences and Veterinary Medicine (UASVM) Cluj-Napoca, Mănăştur Street 3-5, 400372 Cluj-Napoca, Romania; alexandru-florin.lupsan@usamvcluj.ro (A.F.L.); cristian.crecan@usamvcluj.ro (C.M.C.); 4 Department of Pathophysiology, Faculty of Veterinary Medicine, University of Agricultural Sciences and Veterinary Medicine (UASVM) Cluj-Napoca, Mănăştur Street 3-5, 400372 Cluj-Napoca, Romania; denisa.bungardean@usamvcluj.ro; 5 Department of Clinics, Faculty of Veterinary Medicine, Iași University of Life Sciences “Ion Ionescu de la Brad”, 700490 Iaşi, Romania; mihai.musteata@iuls.ro (M.M.); paula.pasca@iuls.ro (P.M.P.); 6 Department of Clinical Sciences, Faculty of Veterinary Medicine, University of Agricultural Sciences and Veterinary Medicine (UASVM) Cluj-Napoca, 3-5 Mănăştur Street, 400372 Cluj-Napoca, Romania; sorin.marza@usamvcluj.ro (S.M.M.); robert.purdoiu@usamvcluj.ro (R.C.P.); ionel.papuc@usamvcluj.ro (I.P.); radu.lacatus@usamvcluj.ro (R.L.); teodora-sonia.patrichi@student.usamvcluj.ro (T.S.P.); 7 Department of Public Health and Food Hygiene, Faculty of Veterinary Medicine, University of Agricultural Sciences and Veterinary Medicine (UASVM) Cluj-Napoca, 400372 Cluj-Napoca, Romania; carolinelacatus@yahoo.com (C.M.L.); luciana.rus@usamvcluj.ro (L.C.P.); 8 Plastic Surgery Department, University of Medicine and Pharmacy, 8 Victor Babeş Street, 400012 Cluj-Napoca, Romania; irmatei@yahoo.com; 9 Plastic Surgery Clinic, Spitalul Clinic de Recuperare, 46-50 Viilor Street, 400347 Cluj-Napoca, Romania; 10 Horticulture and Landscaping Department, Faculty of Horticulture and Business in Rural Development, University of Agricultural Sciences and Veterinary Medicine (UASVM) Cluj-Napoca, 3-5 Mănăștur Street, 400372 Cluj-Napoca, Romania; cristian.sisea@usamvcluj.ro; 11 Viticulture and Oenology Department, Faculty of Horticulture and Business in Rural Development, University of Agricultural Sciences and Veterinary Medicine (UASVM) Cluj-Napoca, 3-5 Mănăștur Street, 400372 Cluj-Napoca, Romania; claudiu.bunea@usamvcluj.ro (C.I.B.); anamaria.calugar@usamvcluj.ro (A.C.); 12 Department of Environmental Engineering and Protection, Faculty of Agriculture, University of Agricultural Sciences and Veterinary Medicine (UASVM) Cluj-Napoca, 3-5 Mănăştur Street, 400372 Cluj-Napoca, Romania; ioan.mag@usamvcluj.ro; 13 Bioflux SRL, 54 Ceahlău Street, 400488 Cluj-Napoca, Romania; 14 Doctoral School of Engineering, University of Oradea, 1 Universităţii Street, 410087 Oradea, Romania; 15 Department of Internal Medicine, Faculty of Veterinary Medicine, University of Agricultural Sciences and Veterinary Medicine (UASVM) Cluj-Napoca, Mănăştur Street 3-5, 400372 Cluj-Napoca, Romania; sofia.daradics@usamvcluj.ro; 16 Laboratory of Chromatography, Advanced Horticultural Research Institute of Transylvania, Faculty of Horticulture and Business for Rural Development, University of Agricultural Sciences and Veterinary Medicine (UASVM) Cluj-Napoca, 400372 Cluj-Napoca, Romania

**Keywords:** heavy metal bioaccumulation, equine bioindicators, environmental pollution monitoring, hematological biomarkers, multi-matrix analysis

## Abstract

Environmental contamination with heavy metals, resulting from industrialization, urbanization, and agricultural intensification, poses serious ecological and health risks. Horses, due to their grazing behavior and close association with human environments, serve as reliable sentinel species for assessing environmental pollution. This study aimed to evaluate the bioaccumulation of heavy metals and trace elements in different biological matrices of horses—blood, hair, hooves, and synovial fluid—and to investigate their relationship with hematological biomarkers as indicators of physiological stress. Samples were collected from horses raised in anthropogenically influenced areas and analyzed using inductively coupled plasma mass spectrometry (ICP–MS). Hematological parameters were determined with an automated analyzer to assess systemic effects. The results revealed significant variations in metal concentrations among matrices, with keratinized tissues reflecting long-term exposure, while blood and synovial fluid indicated recent contamination. Correlations between elevated metal levels and altered hematological values suggested oxidative stress and adaptive physiological responses. These findings demonstrate the value of multi-matrix biomonitoring in evaluating both environmental quality and animal health. Horses effectively reflect the cumulative impact of heavy metal exposure, supporting their role as bioindicators within a One Health framework that links environmental, animal, and human well-being.

## 1. Introduction

Over the past century, rapid industrialization, urbanization, and intensive agricultural practices have dramatically increased environmental pollution. Among the resulting contaminants, heavy metals are of particular concern due to their persistence, bioaccumulative nature, and toxicity to human and other animals’ health throughout ecosystems [1]. Although certain metals such as zinc (Zn), copper (Cu), and iron (Fe) are essential for biological functions, their excessive accumulation can disrupt metabolic processes and cause toxic effects [2].

The mobility and bioavailability of heavy metals in soil are mainly controlled by pH, organic matter, and cation exchange capacity (CEC). Low pH increases the solubility and mobility of metals such as lead (Pb), cadmium (Cd), and zinc (Zn), while alkaline conditions favor their precipitation as insoluble compounds, reducing uptake [3,4]. Organic matter and humic substances bind and chelate metal ions, limiting their transport [5]. Soils with high CEC, rich in clay and humic components, retain metals more effectively, although competing cations (Ca^2+^, Mg^2+^, K^+^) can displace them and increase bioavailability [6]. Understanding these processes is essential for assessing metal behavior and developing soil remediation strategies [7].

Water contamination by heavy metals strongly influences their bioavailability and transfer through the food chain, threatening ecosystem integrity and food safety [8,9]. In aquatic environments, metals occur as free ions or complexed species, with their mobility and uptake determined by pH, redox potential, and dissolved organic matter [8,9]. Highly bioavailable metals such as cadmium (Cd^2+^), lead (Pb^2+^), and mercury (Hg^2+^) are easily absorbed by aquatic organisms, bioaccumulating across trophic levels [2] and threatening ecosystem [2] and food safety [8,9]. Maintaining high water quality standards is therefore crucial for safeguarding animal health [2] and food safety [10].

Heavy metal exposure adversely affects animal and human health by disrupting metabolic, enzymatic, and immune functions. Lead (Pb), cadmium (Cd), and arsenic (As) cause oxidative stress and protein disruption, leading to anemia, organ damage, and neurological disorders [11,12,13,14,15]. In grazing animals, contamination from forage, soil, and water results in metal bioaccumulation and reduced productivity [2,15]. Among domestic species, horses are particularly valuable for such monitoring due to their sensitivity to environmental pollutants [2,15]. Beyond environmental exposure, feed contamination represents another important source of heavy metal intake in horses [14,16]. Pollutants such as lead (Pb), cadmium (Cd), arsenic (As), and mercury (Hg) can enter concentrated feed through contaminated raw materials (e.g., cereals, oilseeds, mineral supplements) and processing steps including milling, storage, or packaging [14,16]. Chronic ingestion of contaminated feed may lead to neurological, hepatic, renal, and hematological alterations [15]. To reduce these risks, regular quality control and the use of adsorbents such as zeolites are recommended to limit metal bioavailability and accumulation [17].

Trace elements such as zinc (Zn), iron (Fe), and copper (Cu), which are essential for normal physiological processes, are naturally absorbed by pasture plants or supplemented through hay and concentrates [18]. Their concentrations in grazing animals depend on soil composition, plant species, forage maturity, and preservation methods, but excessive accumulation can become toxic [2,19,20]. Environmental contamination by heavy metals arises from both natural and anthropogenic sources, including industrial effluents, fertilizer application, and urban runoff [14,18]. Once introduced into the food chain, these metals bioaccumulate in tissues, increasing in concentration at higher trophic levels [12]. Animals serve as effective bioindicators of environmental pollution by heavy metals, as they share habitats with humans and are exposed to the same environmental contaminants [21]. Grazing species are particularly vulnerable to metal exposure through the ingestion of contaminated vegetation, trace amounts of soil, and, in some cases, polluted water sources [22]. Prolonged exposure through multiple pathways results in the accumulation of metals in various tissues [23]. The analysis of biological materials such as soft tissues, urine, hair, whole blood, and serum provides valuable indicators of environmental degradation [23]. Given their proximity to human settlements and shared exposure to pollutants, horses serve as valuable bioindicators of environmental contamination [16]. Monitoring metal accumulation in their tissues and biological fluids provides important insight into environmental quality and potential health risks to both animals and humans [16].

Horses serve as valuable bioindicators of environmental contamination due to their close interaction with human populations, shared habitats, and physiological similarities to humans, particularly in metabolic and organ functions [2,13,14]. As grazing animals, they are continuously exposed to pollutants accumulated in soil, water, and forage, making them effective sentinel organisms for environmental monitoring [2,14]. Assessing heavy metal concentrations in equine biological matrices such as whole blood, serum, hair, and hooves provides insight into both acute and chronic exposure levels [13,14,19,24,25]. While blood reflects short-term contamination, keratinized tissues—particularly hair and hooves—serve as stable, non-invasive indicators of long-term exposure due to their ability to incorporate metals during tissue formation [26,27]. These matrices therefore complement each other in evaluating bioaccumulation patterns and in correlating environmental metal loads with physiological and hematological responses in horses [11,14].

Keratinized tissues, such as hair and hooves, act as long-term biological archives of heavy metal exposure due to their slow growth and low metabolic turnover. Metals bind to sulfhydryl (-SH) groups in keratin proteins during tissue formation, ensuring their stability and persistence over time [28]. Nevertheless, accurate interpretation requires careful sample preparation, as external contamination from dust, soil, or feed can alter measured concentrations [29].

The analysis of hair as a biomarker for evaluating mineral and heavy metal status has been extensively applied across animal species [30]. Hair is considered an advantageous matrix due to its ease of collection, non-invasive sampling, and long-term stability compared to blood or soft tissues [30]. During follicular growth, it acts as a metabolically active structure that incorporates minerals from circulating fluids and blood, while after keratinization, it becomes metabolically inert, preserving the elemental composition from its formation period [31]. Consequently, hair provides an integrated record of prolonged exposure to metals, making it particularly valuable for monitoring environmental contamination. However, factors such as age, breed, hair color, and seasonal growth variations can influence mineral composition, which should be considered when interpreting results [30].

Hair, recognized as an indicator of long-term mineral exposure, reflects cumulative changes in trace element accumulation over time [32]. Investigating correlations between hair and blood mineral concentrations [30], as well as age-related variations, can provide valuable insight into the metabolic distribution of elements in horses [23]. Given that hair mineral concentrations are generally higher than in other tissues, this matrix represents whole-body exposure rather than short-term fluctuations [33,34]. Therefore, hair analysis is particularly suited for monitoring population-level exposure to metals, supporting the establishment of reference values and improving animal health assessment [33,34].

Blood and synovial fluid represent critical biological matrices for assessing recent heavy metal exposure, as they reflect acute contamination and short-term bioavailability [1]. Metals in these fluids bind to proteins, enzymes, and cellular components, influencing their toxicity and biological behavior [9]. However, due to rapid metabolism and renal excretion, blood concentrations can fluctuate over short periods, whereas synovial fluid offers additional insights into the local deposition of metals within joints and their potential involvement in inflammatory and degenerative processes [35].

Exposure to heavy metals inevitably affects blood biomarkers, reflecting the body’s physiological response to toxic interactions [2]. Hematological biomarkers provide a comprehensive assessment of the cumulative effects of multiple pollutants, enabling early detection of health disturbances and prediction of long-term consequences [2]. Given the increasing environmental pressure from industrialization, urbanization, and intensive agriculture, pollution monitoring has become essential for ensuring species health, ecosystem stability, and environmental safety [2].

Hematological biomarkers provide valuable tools for detecting homeostatic alterations caused by heavy metal accumulation in horses [2,24,25]. The hematological profile serves as a sensitive indicator of physiological status, reflecting the organism’s adaptive response to metal-induced stress [19,36,37]. Exposure to toxic metals triggers defense mechanisms that lead to variations in blood cell counts [38], enzymatic activity, and immune function, highlighting the importance of hematological assessment in environmental biomonitoring [23].

The metabolism and age of the animal significantly influence the accumulation and elimination of heavy metals [39]. Younger animals, characterized by higher metabolic rates and faster cell turnover, may absorb metals more efficiently due to increased intestinal permeability and elevated physiological demands for essential trace elements [39]. However, they generally exhibit more effective detoxification and excretory mechanisms, reducing long-term metal retention. In contrast, older animals, having undergone prolonged exposure, tend to show higher bioaccumulation levels—particularly in keratinized tissues and bone—where metals such as lead (Pb) and cadmium (Cd) can persist for years [39]. Age-related declines in hepatic and renal detoxification further contribute to metal accumulation, underscoring the importance of considering metabolic factors when assessing environmental contamination and animal health [40].

The effectiveness of environmental monitoring depends on selecting appropriate biological matrices that reflect both contaminant properties and physiological processes [40]. Heavy metals accumulate differently depending on factors such as age and metabolic rate—young animals may detoxify contaminants more efficiently, whereas older individuals tend to retain higher levels over time [40]. Advances in analytical techniques, including inductively coupled plasma mass spectrometry (ICP–MS) and biomarker assays, have greatly improved the precision of exposure assessments, supporting risk evaluation and pollution control strategies [40]. Integrating multiple biological (e.g., blood, urine, hair, hooves) and environmental (e.g., soil, water, air) matrices provides [2] a comprehensive view of exposure dynamics, enhancing risk assessment and environmental protection within a One Health framework [41].

The primary objective of this study was to assess the bioaccumulation of heavy metals and trace elements in different biological matrices of horses in order to determine the impact of environmental contaminant exposure on their health. It is hypothesized that chronic exposure of horses to heavy metals from soil, water and feed leads to their accumulation in various biological matrices (blood, hair, hooves, biological fluids). Furthermore, it is expected that keratinized tissues (hair, hooves) provide a more accurate reflection of long-term exposure compared to blood samples, which indicate recent contamination.

## 2. Materials and Methods

### 2.1. Study Site and Samping Design

A total of 390 representative samples were analyzed across eight categories: soil (48), grass (48), hay (48), concentrate feed (24), water (24), mane and tail hair (66), hoof wall and sole (66), and biological fluids (33 serum and 33 synovial fluid samples). These originated from 36 locations distributed across three contaminated zones and one control area in northwestern Romania (Maramureș County), historically impacted by non-ferrous metal mining. In total, 576 field subsamples were initially collected in triplicate and subsequently composited into 390 analytical samples. Variations in sample number reflect matrix availability concentrates were collected only in the control area, and synovial fluid only from a limited number of horses ensuring representative coverage across environmental and biological compartments.

The contaminated areas included in this study were rigorously selected based on extensive environmental monitoring evidence and well-documented historical records of severe heavy metal pollution in the Baia Mare mining region. This region is internationally recognized as one of the most severely affected areas in Europe as a consequence of decades of intensive non-ferrous mining and metallurgical operations. Multiple scientific investigations and governmental reports have consistently demonstrated critical exceedances of Pb, Zn, Cu and Cd concentrations in soil, vegetation, water sources and atmospheric deposits surrounding former industrial sites, confirming the persistence of long-term environmental contamination and justifying their inclusion in the present study.

Zone I included the vicinity of the former tailing’s ponds at Bozânta Mare, Săsar, and Nistru, located approximately 6–10 km from the former Aurul and Romplumb industrial complexes. Zone II encompassed the former mining areas of Herja, Ilba, Șuior, and Nistru, as well as the UP Central Flotation Facility at Bozânta Mare, which remain characterized by the persistence of heavy metal residues (Pb, Zn, Cu, Cd). Zone III represented the control area, located in Tîrlișua (Bistrița-Năsăud County), approximately 70 km south of Baia Mare, where no mining or industrial activities are present, serving as a reference site for baseline contamination assessment. Three replicate samples were collected from each site for every matrix to ensure reproducibility and analytical consistency. To enable comparison and evaluate both environmental and biological exposure gradients, the control area of Tîrlișua was included due to its lack of known heavy metal sources and the absence of prior contamination studies. The complete inventory of samples, including codes and collection dates, is provided in the Supplementary Materials (Tables S1–S5). Precise geospatial coordinates for all sampling points were recorded using a Garmin eTrex 32× TopoActive Europe 2.2 handheld GPS device (Garmin Ltd., Olathe, KS, USA).

### 2.2. Sampling of Environmental and Biological Matrices

#### 2.2.1. Mane and Tail Hair Sample Collection

Mane and tail hair samples were collected during February–March 2025. For each animal, approximately 5–10 g of hair was obtained from both the middle section of the mane and the base of the tail, using cleaned stainless-steel scissors and powder-free nitrile gloves to prevent external contamination. Hair was cut as close as possible to the skin, avoiding areas exposed to sweat, dust, or feed residues. Immediately after collection, each sample was placed in a labeled polyethylene bag indicating the sample code (corresponding to the individual horse), collection site, date, and owner identification. All samples were transported to the laboratory on the same day and stored in a clean, dry environment until preparation for further analysis. Detailed information on sampling locations, horse ownership, and collection dates is provided in Supplementary Table S1.

#### 2.2.2. Hoof Sample Collection

Hoof samples were collected during routine farriery or trimming procedures. For each individual, approximately 5–10 g of hoof trimmings were obtained from the dorsal wall, sole, or frog using pre-cleaned stainless-steel tools and powder-free nitrile gloves to prevent external contamination. Samples were collected only from clean, dry areas of the hoof, avoiding contact with mud, manure, or bedding materials. Immediately after collection, each sample was placed in a labeled polyethylene bag indicating the sample code, collection site, date, and horse owner. All samples were transported to the laboratory on the same day and stored at 4 °C in a clean, dry environment until preparation for further analysis. Detailed information on sampling locations, ownership, and collection dates is presented in Supplementary Table S2.

#### 2.2.3. Serum Sample Collection

Sampling was conducted in October 2025 following the established study design. Blood was drawn from the jugular vein using vacuum collection tubes without anticoagulant, ensuring aseptic conditions and minimal stress to the animals. For each horse, approximately 10 mL of blood was collected and allowed to clot at ambient temperature. To maintain sample integrity, all samples were kept under cold chain conditions (4 °C) immediately after collection and transported to the laboratory within 24 h. Each serum sample was labeled with a unique identification code indicating the sampling site, owner number, and collection date. Detailed information on sampling zones, horse owners, and collection chronology is presented in Supplementary Table S3.

#### 2.2.4. Synovial Fluid Sample Collection

Sampling was conducted in October 2025 following standard veterinary procedures. Synovial fluid was aspirated using sterile 18G or 20G hypodermic needles attached to sterile syringes, from selected joints identified according to anatomical landmarks. Prior to sampling, the site was disinfected using a three-step protocol: cleansing with 2% povidone-iodine, rinsing with 70% ethanol, and covering with a sterile drape. For each horse, 2–5 mL of synovial fluid was collected, discarding the initial 0.5 mL to minimize contamination from periarticular tissues. Samples showing visible blood contamination were excluded. Each specimen was immediately transferred to a labeled sterile tube indicating the sample code, location, collection date, and owner identification and stored at 4 °C until delivery to the laboratory. Detailed information regarding sampling sites, owners, and collection chronology is provided in Supplementary Table S4.

#### 2.2.5. Water Sample Collection

Water samples were collected from various horse drinking sources, including troughs, wells, ponds, and streams. Sampling was performed in April–June 2025 following standardized clean procedures to prevent trace metal contamination. Prior to collection, all containers and equipment were acid-washed and rinsed with deionized water. At each site, samples were collected using pre-cleaned polyethylene bottles according to EPA Method 1669 (“Clean Hands/Dirty Hands” technique). For surface waters (ponds and streams), samples were taken at a depth of approximately 15–30 cm below the surface, avoiding sediment disturbance. For wells and troughs, water was first mixed to ensure homogeneity before collection. Each sample (approximately 500 mL) was immediately acidified to pH < 2 with ultrapure nitric acid (HNO_3_) to preserve dissolved metal species. Bottles were tightly sealed, labeled with the sample code, site, and collection date, and stored in a cooler at 4 °C during transport to the laboratory. Detailed information on sampling locations, environmental characteristics, and collection chronology is provided in Supplementary Table S5.

#### 2.2.6. Green Grass and Dried Hay Sample Collection

Dried hay and green grass samples were collected from representative grazing and hay production areas, selected based on accessibility and the absence of contamination sources such as pesticides, fertilizers, or animal waste. At each site, GPS coordinates and environmental conditions (vegetation type, soil condition, and weather) were recorded. To prevent contamination, sterile gloves and clean stainless-steel scissors were used. Approximately 500 g of fresh grass were cut from multiple points selected randomly within a 100 × 100 m sampling grid using GPS coordinates, placed in labeled polyethylene bags, and transported in coolers with ice packs. If immediate processing was not possible, samples were frozen at −20 °C. Dried hay samples (≈500 g) were taken from the inner layers of different bales to minimize surface contamination; stored in breathable paper bags; and kept in dry, ventilated conditions before laboratory processing. In the laboratory, grass samples were either frozen at −20 °C or oven-dried at 60 °C to constant weight, while hay samples were stored under cool, dry conditions until analysis. For moisture determination, samples were oven-dried at 105 °C for 24 h, and the percentage of moisture was calculated based on weight loss. Detailed information on sample codes, collection sites, and dates is provided in Supplementary Table S6.

#### 2.2.7. Concentrate Sample Collection

Concentrate feed samples were collected from feed storage areas and feeding troughs used for the same horses included in this study. Sampling was performed in February 2025, immediately after feed distribution or during storage inspection, to ensure representativeness and prevent cross-contamination. At each site, approximately 500 g of concentrate feed were collected from three different points within each feed batch (top, middle, and bottom layers) using pre-cleaned plastic scoops and powder-free nitrile gloves. Samples were transferred into labeled polyethylene bags indicating the sample code, collection site, date, and owner identification. To prevent moisture absorption and contamination, bags were tightly sealed and stored in a cool, dry environment (4–8 °C refrigeration chamber with controlled humidity < 50%) before transport to the laboratory. All samples were delivered to the laboratory on the same day of collection and stored at room temperature in a clean, ventilated area until processing. Detailed information on sampling sites, ownership, and collection chronology is provided in Supplementary Table S7.

#### 2.2.8. Soil Sample Collection

Soil samples were collected from a total of four study zones—three located in historically contaminated mining areas and one representing an uncontaminated control area—during the spring–summer of 2025 using a pragmatic sampling approach, meaning that sampling locations were chosen based on safe access, landowner permission, and suitable ground conditions to ensure representative coverage of each area (Bora et al., 2023) [42]. At each site, about 0.5 kg of topsoil (0–10 cm) was obtained as a composite sample from three sub-points within a 100 × 100 m area. Sampling sites were selected based on accessibility and with landowner authorization. Soil was collected with a PVC corer and plastic trowel (ISO 11464:1994) [43], avoiding large aggregates and debris. In the laboratory, samples were cleaned of roots and stones, homogenized, and prepared for analysis. Geographic and environmental details of sampling sites are presented in Supplementary Table S8.

### 2.3. Sample Preparation, Digestion, and Analytical Determination of Metals

#### 2.3.1. Sample Preparation

##### Mane and Tail Hair Samples

Hair samples (mane and tail) were initially rinsed with ultrapure deionized water to remove loosely attached surface particles. Subsequently, they were immersed in a 1:100 (*v*/*v*) Triton X-100 solution (Sigma-Aldrich, St. Louis, MO, USA) and sonicated for 10 min at 30 °C to eliminate lipids, sweat residues, and dust contaminants. The samples were then rinsed three times with ultrapure water, followed by two washes with analytical-grade acetone to ensure complete degreasing. Cleaned hair was air-dried for 48 h in a dust-free environment, cut into 1–2 cm segments using pre-cleaned stainless-steel scissors, and homogenized. To prevent mineral loss prior to analysis, the samples were additionally washed with methanol and air-dried. The processed samples were stored in acid-washed, pre-labeled polyethylene containers within a desiccator until digestion.

##### Hoof Samples

Collected hoof trimmings were cleaned to eliminate any external contamination. The samples were rinsed with ultrapure water, brushed mechanically with a stainless-steel tool, and sonicated in a 1:100 (*v*/*v*) Triton X-100 solution for 10 min at 30 °C. This was followed by two acetone rinses and three deionized water washes. After cleaning, the samples were air-dried for 48 h, finely ground using a Retsch 110 automatic mill (Merck KGaA, Darmstadt, Germany), sieved through a 2 mm mesh, and stored in pre-labeled polyethylene vials in a desiccator until further analysis.

##### Serum Samples

Blood samples were centrifuged at 3000× *g* for 10 min at 4 °C to separate the serum fraction. The supernatant was carefully transferred into acid-washed polypropylene tubes and immediately stored at −20 °C until digestion. Each serum sample was thawed only once prior to mineralization to minimize degradation and prevent adsorption of trace metals.

##### Synovial Fluid Samples

Synovial fluid samples were centrifuged at 3000× *g* for 10 min at 4 °C to remove cellular debris and insoluble material. The supernatant was filtered through a 0.45 µm Whatman 42 membrane filter. For protein precipitation and matrix simplification, 10% trichloroacetic acid (TCA) was added in a 1:5 (*v*/*v*) ratio, and the mixture was incubated at 4 °C for 30 min. The clear supernatant was filtered again through a 0.22 µm syringe filter, transferred into acid-washed polypropylene vials, and stored at −20 °C until digestion.

##### Water Samples

Water samples collected from various drinking sources (wells, troughs, ponds, and streams) were filtered through 0.45 µm PTFE membrane filters to remove suspended solids. Immediately after filtration, each sample was acidified with ultrapure nitric acid (HNO_3_, Suprapur®, Merck KGaA, Darmstadt, Germany) to adjust the pH below 2, thereby preventing metal precipitation or adsorption onto the container walls. The acidified samples were stored at 4 °C in pre-cleaned, labeled polyethylene bottles until analysis.

##### Green Grass and Dried Hay Samples

Vegetation samples were inspected to remove foreign material and washed sequentially with tap water, deionized water, and finally ultrapure water to eliminate external dust or adhering soil particles. Green grass samples were oven-dried at 105 °C until constant weight (72–96 h), whereas hay samples were dried at 60 °C for 48–72 h to preserve their structural integrity. After drying, the samples were ground using a Retsch 110 automatic mill (Merck KGaA, Darmstadt, Germany), sieved through a 2 mm nylon mesh, and homogenized. The resulting powdered material was stored in acid-washed polyethylene containers within a desiccator until digestion.

##### Concentrate Feed Samples

Representative portions of concentrate feed were collected from the upper, middle, and lower sections of each batch to ensure sample uniformity. The samples were homogenized, oven-dried at 60 °C to constant mass, and finely milled using a stainless-steel grinder. The ground material was passed through a 2 mm sieve to obtain a homogeneous powder and stored in clean, dry, labeled polyethylene containers prior to analysis.

##### Soil Samples

Soil samples were air-dried at room temperature for 72 h, followed by oven-drying at 105 °C until constant weight using a Binder FD 53 oven (BINDER GmbH, Tuttlingen, Germany). The dried material was sieved through a 2 mm nylon mesh to remove stones, roots, and plant residues, then homogenized and finely pulverized using an agate mortar and pestle. The processed soil samples were transferred into pre-cleaned, acid-washed HDPE containers, labeled, and stored in a desiccator at ambient temperature until digestion.

#### 2.3.2. Sample Digestion

Sample digestion was performed using a Milestone START D Microwave Digestion System (Milestone S.r.L, Sorisole, Italy), following the manufacturer’s standard programs optimized for each matrix type (Table S9). Approximately 0.5 g of solid samples (soil, hay, grass, concentrate feed, hair, hoof) or 5 mL of liquid samples (serum, synovial fluid, water) were placed in Teflon vessels and treated with 6 mL HNO_3_ (65%) and 2 mL H_2_O_2_ (30%) (both Suprapur^®^, Merck KGaA, Darmstadt, Germany). The digestion programs corresponded to the following Milestone methods: DG_EN-12 (Soil), DG_EN-05 (Grass), DG_EN-18 (Wastewater), DG-FO-61/DG_AG-04 (Cereals/Maize), DG-CL-10 (Hair), DG-CL-02 (Animal tissue), and DG-CL-03 (Blood). The system operated at a maximum temperature of 200–230 °C, pressure up to 800 psi, and full power (100%). Total digestion, including cooling, lasted approximately 35–55 min, depending on the matrix.

After cooling, digests were quantitatively transferred to 50 mL volumetric flasks, diluted to volume with ultrapure water (18.2 MΩ·cm, Milli-Q), and filtered through 0.45 µm PTFE membranes. Reagent blanks were prepared for each batch, and certified reference materials (CRMs) of similar matrices were processed in parallel to verify accuracy. All digests were stored at 4 °C in acid-washed polyethylene vials until ICP–MS analysis.

#### 2.3.3. Analytical Determination of Metals

The quantitative determination of trace and heavy metals (Cu, Zn, Pb, Cd, Ni, Co, As, Cr, and Hg) was performed using an ICP–MS system (Thermo Scientific iCAP Q, Bremen, Germany). Instrumental parameters and data acquisition settings are summarized in Table S10, and analytical performance characteristics (LoD, LoQ, and BEC) are listed in Table S11. The instrument operated in standard mode with high-purity argon (Ar 5.0) as plasma gas and helium (He 6.0) in the collision cell to minimize spectral interferences. Calibration was carried out daily using multi-element standards (Merck Certipur®, Merck KGaA, Darmstadt, Germany) between 0.1 and 100 µg/L, achieving R^2^ > 0.999. Internal standards (Rh, In, Re) were continuously applied to correct for matrix and instrumental drift.

Method accuracy and precision were verified using certified reference materials (CRMs) representative of each matrix, with recoveries of 90–110% and uncertainties below 20%. Detection limits ranged from 0.006 µg/L (As) to 1.607 µg/L (Cr), depending on the matrix. Analytical quality was ensured through reagent blanks, duplicates, and CRMs in each batch, achieving reproducibility within ±5%. Results were reported as µg/L for liquids and mg/kg (dry weight) for solids.

### 2.4. Ethical Considerations

All procedures complied with European Directive 2010/63/EU and Romanian Law No. 43/2014 regarding animal experimentation. The study protocol “Combined Effects of Environmental and Dietary Factors on Heavy Metal Bioaccumulation in Horses and Health Risk Assessment” received ethical approval from the Bioethics Committee of the University of Agricultural Sciences and Veterinary Medicine of Cluj-Napoca (Decision No. 491/21.01.2025 for non-invasive sampling; Decision No. 530/03.10.2025 for invasive procedures). Invasive sampling (blood and synovial fluid) was carried out by licensed veterinarians, and written owner consent was obtained prior to sample collection.

### 2.5. Statistical Analysis

Statistical analyses were performed using IBM SPSS Statistics v29.0 and GraphPad Prism v10.0. Data normality was tested using the Shapiro–Wilk test and homogeneity of variances with Levene’s test. Depending on distribution, differences among zones were assessed using one-way ANOVA with Tukey post hoc, or Kruskal–Wallis with Dunn’s multiple comparisons for non-parametric data. Correlations between environmental and biological matrices were evaluated using Pearson or Spearman coefficients. Results are reported as mean ± SD, and *p* < 0.05 was considered statistically significant. The Cumulative Pollution Index (CPI) was calculated to provide an integrated measure of metal contamination across environmental and biological matrices. CPI was computed based on the ratio between the concentration of each metal (Ci) and the corresponding reference value obtained from the control zone (Cref), according to the following formula:

$$CPI=\sum (\frac{C_{i}}{C_{ref}})$$

where Ci represents the measured concentration in contaminated zones and Cref the baseline concentration from the control area. CPI values > 1 indicate contamination above natural background levels, whereas values < 1 indicate lower or comparable concentrations. This index allows comparison across matrices with different scales, integrating environmental and biological data into a single interpretation framework.

NotebookLM AI Assistant was used solely to improve clarity and structure during manuscript preparation, specifically for text refinement, organization, and linguistic optimization in the Introduction section. No AI tool was used to generate scientific content, analyze data, or influence the research findings. All AI-assisted suggestions were manually reviewed, edited, and validated by the authors, who take full responsibility for the final manuscript.

## 3. Results

Heavy metal concentrations varied across matrices. Environmental samples (soil, water, forage and feed) showed the highest levels in contaminated zones, while biological matrices (hair, hoof, serum and synovial fluid) demonstrated corresponding differences among zones. Keratinized tissues (hair and hoof) exhibited the greatest accumulation potential, followed by serum and synovial fluid, which presented lower concentration values.

### 3.1. Heavy Metal Profiles in Equine Mane and Tail Hair from Polluted and Control Areas

The determination of heavy metal concentrations (Cu, Zn, Pb, Cd, Ni, Co, As, Cr, Hg) in equine mane and tail hair showed clear differences among the studied zones (Table 1). The highest concentrations were recorded in the mining and tailings areas, while the control zone presented substantially lower values. Copper (Cu) ranged from 15.24 to 18.91 mg/kg in the mining area and 10.18–15.08 mg/kg in the tailings zone, compared with only 1.01–1.12 mg/kg in the control group. Differences among zones were statistically significant (Kruskal–Wallis, *p* = 0.021).

Zinc (Zn) showed the highest concentrations in the mining zone (171.55–186.60 mg/kg), intermediate levels in the tailings area (139.52–170.38 mg/kg), and markedly lower values in the control (48.56–51.36 mg/kg). Differences among groups were statistically significant (Kruskal–Wallis, *p* = 0.021) (Table 1). Lead (Pb) followed a similar distribution, with 7.09–7.58 mg/kg in the mining zone and 4.17–6.11 mg/kg in the tailings zone, compared to 1.32–1.49 mg/kg in the control. Group differences were significant (*p* = 0.023), indicating a clear contamination gradient.

Cadmium (Cd) ranged from 0.77 to 1.41 mg/kg in contaminated zones and 0.35–0.36 mg/kg in the control area, with statistically significant differences among groups (*p* = 0.038) (Table 1). Nickel (Ni) showed levels of 0.42–0.66 mg/kg in contaminated sites and 0.16 mg/kg in the control, also with significant variation among zones (*p* = 0.016). Cobalt (Co) concentrations were low overall (0.40–0.67 mg/kg in contaminated areas; 0.04 mg/kg in the control) and not statistically significant (*p* = 0.245), indicating greater variability and limited discriminatory value.

Arsenic (As) ranged from 0.54 to 1.95 mg/kg in contaminated zones and 0.06–0.08 mg/kg in the control area, with significant differences among groups (*p* = 0.046) (Table 1). Chromium (Cr) concentrations were 0.56–1.01 mg/kg in contaminated areas and 0.12–0.13 mg/kg in the control, showing borderline significance (*p* = 0.068). Mercury (Hg) was below detection limits (BLD) in all samples. Comparative analysis between mane and tail hair showed no significant differences for any of the analyzed metals (*p* > 0.05), indicating uniform distribution within hair matrices (Table 1). Spearman correlation analysis showed negative correlations between metal concentrations and exposure gradient for most elements.

### 3.2. Heavy Metal Profiles in Equine Hooves from Polluted and Control Areas

Heavy metal concentrations in equine hoof samples varied significantly among exposure zones (Table 2). Mining and tailings areas showed higher levels of Cu, Zn, Pb, Ni, and Co compared with the control (*p* < 0.05), while Cd, As, Cr, and Hg were below detection limits. Copper (Cu) reached 11.00 mg/kg in mining sites and 4.80–7.60 mg/kg in tailings areas, versus 2.67–2.83 mg/kg in the control (*p* = 0.020). Differences between hoof wall and sole were also significant (*p* = 0.0008), with consistently higher concentrations in the wall. Zinc (Zn) concentrations were highest in the mining zone (144.10–176.77 mg/kg), intermediate in the tailings area (108.68–131.93 mg/kg), and lowest in the control (50.63–75.06 mg/kg) (Table 2). One-way ANOVA indicated significant differences among the three zones (*p* = 0.0005). Lead (Pb) concentrations were higher in the mining (5.65–6.60 mg/kg) and tailings zones (3.60–6.60 mg/kg) compared with the control (1.12–1.41 mg/kg) (Table 2). Differences among zones were statistically significant (*p* = 0.001), and values were significantly higher in the hoof wall than in the sole (*p* = 0.001).

Cadmium (Cd) was below detection limits (BDLs) in all hoof samples (Table 2). Nickel (Ni) ranged from 1.53 to 2.28 mg/kg in the mining zone and 0.85–1.60 mg/kg in the tailings zone, while control samples were below 0.60 mg/kg. Differences in Ni concentrations among the three study zones were statistically significant (*p* = 0.021). However, no significant differences were found between the hoof wall and sole within zones, indicating a uniform metal distribution and steady-state accumulation dynamics. Cobalt (Co) levels were low overall (0.41–0.74 mg/kg in polluted areas; 0.09–0.12 mg/kg in controls), and differences were statistically significant (*p* = 0.0207), suggesting limited accumulation capacity and a stronger dependence on physiological regulation than environmental exposure. Arsenic (As), chromium (Cr), and mercury (Hg) were below detection limits (BDLs) in all hoof samples across all study zones (Table 2). Spearman correlation analysis showed negative correlations for most metals, indicating a consistent decrease in concentrations from mining and tailings areas toward the control region.

### 3.3. Heavy Metal Profiles in Equine Serum from Polluted and Control Area

The determination of heavy metal concentrations in equine serum samples showed clear differences among exposure zones (Table 3). Copper (Cu), zinc (Zn), nickel (Ni), and cobalt (Co) were higher in horses from the mining and tailings areas compared with the control group. In contrast, lead (Pb), cadmium (Cd), arsenic (As), chromium (Cr), and mercury (Hg) were below the limit of detection (LOD) in all serum samples. Copper (Cu) concentrations were highest in the mining zone (8.93–10.23 µg/L), intermediate in the tailings zone (5.28–7.60 µg/L), and lowest in the control (2.68 µg/L) (Table 3). Kruskal–Wallis testing showed no statistically significant differences among zones (H = 2.58, *p* = 0.108).

Zinc (Zn) followed a similar pattern, with mean values of 772–801 µg/L in mining sites, 661–766 µg/L in tailings areas, and approximately 600 µg/L in the control group. Although differences among zones were not statistically significant (*p* > 0.05), the slightly higher concentrations observed in polluted areas may suggest a potential trend related to environmental mobility and dietary exposure, which warrants further investigation. Although the Kruskal–Wallis test did not show significant differences (H = 2.84, *p* = 0.124), Zn concentrations were higher in polluted zones compared with the control. The high affinity of Zn for metalloproteins (e.g., zinc finger transcription factors, metallothioneins) may contribute to its incorporation into circulating pools; however, based on the present data, Zn cannot be considered a statistically reliable marker of exposure, even if a biological trend is suggested.

**Table 2:** Heavy Metal Concentrations (Cu, Zn, Pb, Cd, Ni, Co, As, Cr, Hg) in Equine Hoof Samples from Distinct Environmental Exposure Zones: Comparative Data from Former Mining, Tailings, and Control Areas (mg/kg FM).

Sample Codes/Heavy Metals	Cu	Zn	Pb	Cd	Ni	Co	As	Cr	Hg	Spearman’s r	*p*-Value
Zone I encompass the areas of the former tailing’s ponds at Bozânta Mare, Săsar, and Nistru											
HF-W-TMBTP-O1	7.43 ± 0.16 ^c^	131.93 ± 6.12 ^b^	4.70 ± 0.18 ^a^	<LOD	0.85 ± 0.10 ^e^	0.50 ± 0.02 ^h^	<LOD	<LOD	<LOD	−0.90	0.037
HF-S-TMBTP-O1	5.40 ± 0.10 ^d^	110.33 ± 3.25 ^b^	3.60 ± 0.20 ^a^	<LOD	0.98 ± 0.10 ^e^	0.41 ± 0.03 ^k^	<LOD	<LOD	<LOD	−0.80	0.104
HF-W-RS-O1	7.60 ± 0.20 ^c^	122.44 ± 3.51 ^b^	4.70 ± 0.23 ^a^	<LOD	1.07 ± 0.13 ^d^	0.53 ± 0.02 ^g^	<LOD	0.32 ± 0.06 ^c^	<LOD	−0.90	0.0048
HF-S-RS-O1	4.80 ± 0.90 ^e^	108.68 ± 6.05 ^b^	3.60 ± 0.23 ^a^	<LOD	1.13 ± 0.13 ^d^	0.46 ± 0.09 ^i^	<LOD	0.23 ± 0.02 ^d^	<LOD	−0.94	0.0039
HF-W-TMN-O1	6.80 ± 0.66 ^d^	128.53 ± 6.66 ^b^	6.60 ± 0.49 ^a^	<LOD	1.53 ±0.20 ^c^	0.56 ± 0.02 ^f^	<LOD	<LOD	<LOD	−0.90	0.037
HF-S-TMN-O1	5.00 ± 1.21 ^d^	108.76 ± 6.57 ^b^	5.60 ± 0.36 ^a^	<LOD	1.60 ± 0.18 ^c^	0.46 ± 0.02 ^j^	<LOD	0.21 ± 0.01 ^e^	<LOD	−0.83	0.0416
Zone II includes the areas of the former mines: Herja, Ilba, Șuior, Nistru, and UP Central Flotation											
HF-W-TMH-O1	9.10 ± 1.18 ^b^	157.65 ± 7.47 ^a^	6.50 ± 0.46 ^a^	<LOD	1.83 ± 0.25 ^b^	0.63 ± 0.03 ^d^	<LOD	0.61 ± 0.15 ^a^	<LOD	−0.94	0.0048
HF-S-TMH-O1	7.60 ± 1.35 ^c^	144.79 ± 12.29 ^a^	5.70 ± 0.53 ^a^	<LOD	1.62 ± 0.23 ^c^	0.52 ± 0.03 ^g^	<LOD	0.52 ± 0.08 ^b^	<LOD	−0.89	0.0188
HF-W-TMH-O2	10.20 ± 1.91 ^a^	169.98 ± 3.99 ^a^	6.40 ± 0.48 ^a^	<LOD	1.95 ± 0.25 ^a^	0.68 ± 0.06 ^c^	<LOD	<LOD	<LOD	−0.9	0.0374
HF-S-TMH-O2	8.10 ± 2.11 ^c^	157.82 ± 9.21 ^a^	5.80 ± 0.48 a	<LOD	1.78 ± 0.23 ^b^	0.56 ± 0.03 ^f^	<LOD	<LOD	<LOD	−0.9	0.0365
HF-W-CI-O1	8.60 ± 1.65 ^b^	167.62 ± 8.53 ^a^	6.30 ± 0.36 ^a^	<LOD	1.58 ± 0.20 ^c^	0.64 ± 0.01 ^d^	<LOD	<LOD	<LOD	−0.9	0.0372
HF-S-CI-O1	7.10 ±1.61 ^c^	147.94 ± 10.73 ^a^	5.60 ± 0.56 ^a^	<LOD	1.73 ± 0.20 ^b^	0.57 ± 0.04 ^f^	<LOD	<LOD	<LOD	−0.92	0.0341
HF-W-CI-O2	11.00 ± 2.00 ^b^	171.11 ± 11.47 ^a^	6.20 ± 0.36 ^a^	<LOD	1.65 ± 0.08 ^b^	0.67 ± 0.03 ^c^	<LOD	<LOD	<LOD	−0.94	0.0064
HF-S-CI-O2	6.70 ± 2.03 ^d^	146.38 ± 13.72 ^a^	5.70 ± 0.46 ^a^	<LOD	1.82 ± 0.19 ^b^	0.56 ± 0.03 ^f^	<LOD	<LOD	<LOD	−0.9	0.0376
HF-W-CȘ-O1	8.80 ± 1.87 ^b^	176.77 ± 9.73 ^a^	6.50 ± 0.46 ^a^	<LOD	2.03 ± 0.34 ^a^	0.69 ± 0.07 ^b^	<LOD	0.19 ± 0.03 ^e^	<LOD	−0.94	0.0054
HF-S-CȘ-O1	6.60 ± 1.87 ^d^	148.19 ± 13.19 ^a^	5.90 ± 0.04 ^a^	<LOD	2.18 ± 0.09 ^a^	0.58 ± 0.01 ^e^	<LOD	<LOD	<LOD	−0.9	0.0374
HF-W-CȘ-O2	9.70 ± 2.33 ^b^	172.49 ± 14.91 l^a^	5.80 ± 0.43 ^a^	<LOD	2.10 ± 0.23 ^a^	0.72 ± 0.03 ^a^	<LOD	0.25 ± 0.06 ^d^	<LOD	−0.94	0.0048
HF-S-CȘ-O2	7.93 ± 2.47 ^c^	144.10 ± 14.85 ^a^	5.80 ± 0.43 ^a^	<LOD	2.23 ± 0.25 ^a^	0.60 ± 0.10 ^d^	<LOD	0.19 ± 0.03 ^e^	<LOD	−0.94	0.0048
HF-W-CȘ-O3	8.10 ± 1.87 ^c^	160.51 ± 17.57 ^a^	6.40 ± 0.46 ^a^	<LOD	2.17 ± 0.23 ^a^	0.74 ± 0.09 ^a^	<LOD	0.26 ± 0.08 ^d^	<LOD	−0.98	0.0051
HF-S-CȘ-O3	6.30 ± 1.92 ^d^	138.02 ± 17.92 ^b^	5.95 ± 0.38 ^a^	<LOD	2.28 ± 0.11 ^a^	0.60 ± 0.14 ^d^	<LOD	0.28 ± 0.14 ^d^	<LOD	−0.9	0.0365
HF-W-TNN-O1	9.50 ± 1.95 ^b^	161.45 ± 17.76 ^a^	6.45 ± 0.21 ^a^	<LOD	1.93 ± 0.18 ^a^	0.58 ± 0.08 ^e^	<LOD	<LOD	<LOD	−0.92	0.0341
HF-S-TNN-O1	7.20 ± 2.33 ^c^	143.75 ± 17.36 ^a^	5.65 ± 0.58 ^a^	<LOD	2.07 ± 0.23 ^a^	0.46 ± 0.02 ^i^	<LOD	<LOD	<LOD	−0.98	0.0051
HF-W-UPCFBM-O1	10.40 ± 2.17 ^a^	164.81 ± 11.28 ^a^	6.25 ± 0.16 ^a^	<LOD	2.12 ± 0.07 ^a^	0.48 ± 0.08 ^h^	<LOD	<LOD	<LOD	−0.94	0.0049
HF-S-UPCFBM-O1	7.80 ± 2.00 ^c^	147.68 ± 13.02 ^a^	5.85 ± 0.69 ^a^	<LOD	2.25 ± 0.31 ^a^	0.43 ± 0.02 ^j^	<LOD	<LOD	<LOD	−0.9	0.0372
Zone III includes the control area											
HF-W-T-O1-O10	2.67 ± 0.55 ^f^	75.06 ± 6.81 ^c^	1.41 ± 0.09 ^b^	<LOD	0.52 ± 0.08 ^f^	0.12 ± 0.02 ^k^	<LOD	<LOD	<LOD	−0.9	0.0374
HF-S-T-O1-O10	2.83 ± 0.55 ^f^	50.63 ± 8.53 ^c^	1.12 ± 0.13 ^b^	<LOD	0.60 ± 0.10 ^f^	0.09 ± 0.01 ^k^	<LOD	<LOD	<LOD	−0.9	0.0375
Kruskal–Wallis H *p*-value (Polluted Area vs. Control Area)	5.34 0.020	15.21 0.0005	29.30 0.001	–	5.33 0.021	5.35 0.0207	–	–	–	–	–
Kruskal–Wallis H *p*-value (Sample Type (Wall vs. Sole)	11.22 0.0008	4.75 0.0293	7.32 0.001	–	0.35 0.56	5.34 0.0208	–	–	–	–	–
Spearman’s r (r^1^) *p*-value (*p*^1^)	0.70 0.0001	−0.17 0.4011	0.867 0.001	–	0.27 0.176	−0.034 0.871	–	–	–	–	–
Spearman’s r (r^2^) *p*-value (*p*^2^)	0.46 0.017	0.25 0.2120	0.433 0.001	–	0.12 0.566	0.034 0.871	–	–	–	–	–
Spearman’s r (r^3^) *p*-value (*p*^3^)	0.462 0.017	−0.44 0.0260	0.969 0.001	–	0.001 0.999	0.190 0.353	–	–	–	–	–
Comparative Analysis of Heavy Metal Levels in Horse Hoof, Including Samples from Control Areas											
Non-polluted area											
Hoof wall specimens harvested from the external surface of the hoof capsule of horses											
Tocci et al. 2017 [27] ppm	5.7	131.5	2.7	–	5.5	–	–	–	–	–	–
Hoof sole specimens harvested from the solar surface of the hoof capsule of horses											
Tocci et al. 2017 [27] ppm	3.6	102.1	1.7	–	1.1	–	–	–	–	–	–
Hoof specimens harvested from the external surfaces of the hoof capsule of horses											
Rueda-Carrillo et al. 2022 [20] µg/g	1.80	79.1	–	–	–	–	–	–	–	–	–
Aragona et al. 2024 [37] mg/kg	0.058	1.077	0.002	–	–	–	–	–	–	–	–
Stachurska et al. 2011 [46] mg/kg	1.10	–	0.39	0.00	–	–	–	1.31	–	–	–

HF = Hoof; W = Wall; S = Sole; TMBTP = Tăuții-Măgherăuș/Bozânta Mare tailings ponds; RS = Recea/Săsar; TMN = Tăuții-Măgherăuș/Nistru; TMH = Tăuții-Măgherăuș/Herja; CI = Cicârlău/Ilba; CȘ = Cavnic/Șuior; UPCFBM = UP Central Flotation/Bozânta Mare; Tîrlișua = control area. O1–O10 = individual owners. Zone I (n = 9), Zone II (n = 27), and Zone III (n = 30) correspond to tailings, mining, and control areas, respectively. Kruskal–Wallis H = non-parametric test for multiple group comparison; Spearman’s ρ = rank correlation coefficient (where ρ represents the Spearman rank correlation coefficient). Different lowercase letters (a, b, c) indicate statistically significant differences (*p* < 0.05, one-way ANOVA followed by Tukey’s HSD test) (Lowercase letters placed above the bars/values denote statistically significant differences between groups, as determined by one-way ANOVA with Tukey’s HSD post hoc test (*p* < 0.05). <LOD = Below Limit of Detection. Negative ρ values indicate decreasing metal concentrations from contaminated to control zones, reflecting inverse correlations with pollution gradients (where ρ represents the Spearman rank correlation coefficient). Limits of quantification (LoQ): Pb—0.231 µg/L; Cd—0.069 µg/L; Co—0.136 µg/L; As—0.743 µg/L; Hg—0.1379 µg/L. These define the lowest quantifiable concentrations with acceptable analytical precision.

Nickel (Ni) and cobalt (Co) showed the most pronounced differences among exposure zones (Table 3). Ni concentrations ranged from 1.53 to 1.83 µg/L in the mining and tailings areas, compared with 0.17 µg/L in the control group. Co concentrations were 0.29–0.37 µg/L in contaminated zones, while all control samples were below the limit of detection (LOD). Kruskal–Wallis testing demonstrated significant differences for both Ni and Co (*p* = 0.001), and Spearman correlation analysis revealed a strong positive correlation between metal concentrations and exposure level (r = 0.907, *p* = 0.001). In contrast, Pb, Cd, As, Cr, and Hg were below detection limits in all serum samples.

Correlation analysis supported the group comparisons (Table 3). Cu and Zn showed moderate correlation coefficients (Spearman r ≈ 0.46, *p* > 0.1), indicating non-significant associations between serum levels and exposure zone. In contrast, Ni and Co exhibited strong positive correlations with contamination level (r = 0.907, *p* = 0.001), consistent with the marked concentration differences among zones. These findings identify Ni and Co as the most discriminative serum indicators of exposure in the studied areas. However, its utility for tracking classical toxic metals is negligible in this context, as these elements were consistently below detection thresholds. Compared to keratinized tissues such as hooves or hair, serum reflects only short-term exposure rather than long-term accumulation, supporting the use of a multi-matrix approach for comprehensive biomonitoring.

### 3.4. Heavy Metal Profiles in Equine Synovial Fluid from Polluted and Control Areas

The determination of heavy metal concentrations in equine synovial fluid revealed a distinct distribution profile compared with other biological matrices (Table 4). Among the analyzed metals, only copper (Cu) and zinc (Zn) were consistently detectable, whereas Pb, Cd, Ni, Co, As, Cr, and Hg remained below the limit of detection (LOD) in all samples. This reflects the low transfer capacity of these metals into the synovial compartment, which is characterized by slower turnover compared with blood circulation.

Copper (Cu) concentrations ranged from 3.37 to 9.07 µg/L across all sites. Although some samples from mining and tailings zones exceeded the control value (5.09 µg/L), several mining samples were lower, indicating considerable variability and preventing a clear exposure gradient. Differences in synovial Cu concentrations between exposure zones were statistically significant (Kruskal–Wallis, H = 5.33, *p* = 0.021), with higher values in mining and tailings areas compared with the control. These findings indicate that synovial fluid can partially reflect environmental exposure to Cu. Zinc (Zn) concentrations varied widely (108.87–394.63 µg/L), with higher values observed in some mining subzones, but the differences among zones were not statistically significant (Kruskal–Wallis, *p* = 0.181). Although elevated Zn levels appeared more frequently in polluted areas, this trend was not supported statistically. The lack of statistical significance may be related to interindividual variability, potentially influenced by factors such as age, physical activity, or inflammatory status of the joints; however, this interpretation should be considered cautiously, as additional controlled studies are needed to confirm these effects.

Pb, Cd, Ni, Co, As, Cr, and Hg were below detection limits in all samples, suggesting limited availability of these metals in the synovial compartment or preferential accumulation in other tissues. Moreover, the slow turnover of synovial fluid may reduce the detectability of long-term exposure signals, as metal concentrations become progressively averaged over time, making synovial fluid a less sensitive matrix for distinguishing spatial exposure differences.

Correlation analysis did not identify a statistically significant association for Cu (r ≈ 0.46, *p* > 0.1). Although a tendency toward higher concentrations in polluted areas was observed, this trend was not strong enough to demonstrate a reliable relationship, likely due to biological variability and regulatory homeostasis. Therefore, Cu cannot be considered a discriminative biomarker for exposure in this study. For Zn, the correlation was weaker (r ≈ 0.38, *p* > 0.1), confirming that despite elevated levels in some samples, Zn does not serve as a robust discriminative marker between exposure zones.

To the best of our knowledge, this study provides the first reported concentrations of heavy metals in equine synovial fluid. Although its sensitivity as a biomonitoring matrix appears limited, the consistent detection of Cu and Zn demonstrates its potential relevance for assessing exposure to essential trace elements. Table 4 shows that synovial fluid did not reveal statistically significant differences among zones, indicating that it is not suitable as a primary biomarker for environmental metal exposure. However, synovial fluid may still provide complementary physiological information when evaluated alongside serum and keratinized matrices, particularly regarding joint metabolic status or inflammation, which could influence metal distribution patterns.

### 3.5. Heavy Metal Profiles in Water Samples from Polluted and Control Areas

The spatial distribution of heavy metals in water showed clear differences among the three study zones: tailing ponds (Zone I), former mining sites (Zone II), and the control area (Zone III). Concentrations were compared with the legal limits for drinking water (HG 102/2022) [47] (Table S19), with summary statistics provided in Table S20. In tailing pond sites (Herja, Recea–Săsar, Bozânta Mare), several metals exceeded regulatory thresholds. Cu ranged from 1.9 to 3.5 mg/L (maximum 5.54 mg/L), above the 2.0 mg/L limit. Pb exceeded the 0.005 mg/L limit in all samples (0.035–0.087 mg/L). Ni reached 0.09–0.11 mg/L, four–five times higher than the permissible 0.020 mg/L. Co levels reached up to 0.17 mg/L. Zn ranged between 3 and 5 mg/L. Cr remained below 0.05 mg/L, and As, Cd, and Hg were below detection limits. Zone II exhibited the highest contamination levels. At Herja, Cu reached up to 8.43 mg/L (mean 7.56–7.93 mg/L), approximately four times higher than the legal limit (2.0 mg/L). At Șuior, Zn remained elevated (4.10–4.85 mg/L), while Ni and Co reached 0.13–0.16 mg/L and ~0.36 mg/L (maximum 0.89 mg/L), respectively. At Ilba and Nistru, Cu ranged from 1.73 to 2.42 mg/L, Zn from 2.5 to 3.6 mg/L, and Pb from 0.019 to 0.046 mg/L. Cr remained between 0.02 and 0.04 mg/L, whereas As, Cd, and Hg were below detection limits. In contrast, control area concentrations were low and within or below legal thresholds: Cu 0.018–0.021 mg/L, Zn 0.011–0.014 mg/L, and Ni 0.018–0.020 mg/L. Pb was slightly elevated (0.012–0.014 mg/L), exceeding the 0.005 mg/L limit by 2–3 times, while Co remained at background levels (0.002–0.003 mg/L). Cr was <0.002 mg/L, and As, Cd, and Hg were undetectable.

A clear gradient in contamination intensity was observed: Zone II > Zone I > Zone III. Systematic exceedances were recorded for Cu and Ni in Zones I–II and for Pb across all zones. The exceedances were generally two to four times higher than the legal limit for Cu, four to eight times higher for Ni, and up to eighteen times higher for Pb. RSD values were mostly below 6% for Cu and Zn, confirming good analytical precision, while higher variability for Pb and Co reflected short-term fluctuations in particulate phases.

### 3.6. Heavy Metal Profiles in Vegetation (Grass and Hay) from Polluted and Control Areas

The spatial distribution of heavy metals in vegetation showed marked differences among the three study zones (Zone I—tailing ponds, Zone II—former mining sites, Zone III—control). Concentrations were evaluated against the maximum permissible limits for feed materials (Directive 2002/32/EC) (Table S21), with summary statistics provided in Table S22. Vegetation from Zone I exhibited elevated levels of Cu, Zn, Pb, and Cd. Cu ranged from 10.6 to 12.8 mg/kg (maximum 14.9 mg/kg), Zn from 118 to 148 mg/kg, and Pb from 1.25 to 2.12 mg/kg in grass and up to 4.04 mg/kg in hay, exceeding the 0.5 mg/kg regulatory background value. Cd concentrations reached 0.27–0.45 mg/kg in grass and 0.20–0.65 mg/kg in hay. Ni and Co occurred at low levels, while As, Cr, and Hg were below detection limits.

**Table 3:** Heavy Metal Concentrations (Cu, Zn, Pb, Cd, Ni, Co, As, Cr, Hg) in Equine Serum Samples from Distinct Environmental Exposure Zones: Comparative Data from Former Mining, Tailings, and Control Areas (µg/L).

Sample Codes/Heavy Metals	Cu	Zn	Pb	Cd	Ni	Co	As	Cr	Hg	Spearman’s r	*p*-Value
Zone I encompass the areas of the former tailing’s ponds at Bozânta Mare, Săsar, and Nistru											
SE-TMBTP-O1	7.54 ± 0.11 ^c^	751.61 ± 12.67 ^k^	<LOD	<LOD	1.53 ± 0.10 ^c^	0.22 ± 0.03 ^c^	<LOD	<LOD	<LOD	–	–
SE-RS-O1	5.28 ± 0.16 ^c^	661.30 ± 11.11 ^l^	<LOD	<LOD	1.40 ± 0.09 ^d^	0.27 ± 0.06 ^c^	<LOD	<LOD	<LOD	–	–
SE-TMN-O1	7.60 ± 0.10 ^c^	766.19 ± 7.16 ^j^	<LOD	<LOD	1.57 ± 0.07 ^c^	0.31 ± 0.03 ^b^	<LOD	<LOD	<LOD	–	–
Zone II includes the areas of the former mines: Herja, Ilba, Șuior, Nistru, and UP Central Flotation											
SE-TMH-O1	9.10 ± 0.15 ^a^	788.67 ± 6.91 ^b^	<LOD	<LOD	1.83 ± 0.12 ^a^	0.35 ± 0.15 ^a^	<LOD	<LOD	<LOD	–	–
SE-TMH-O2	8.93 ± 0.13 ^b^	786.17 ± 11.67 ^c^	<LOD	<LOD	1.78 ± 0.11 ^a^	0.32 ± 0.04 ^b^	<LOD	<LOD	<LOD	–	–
SE-CI-O1	10.23 ± 0.15 ^a^	801.48 ± 3.64 ^a^	<LOD	<LOD	1.57 ± 0.08 ^c^	0.29 ± 0.06 ^c^	<LOD	<LOD	<LOD	–	–
SE-CI-O2	8.10 ± 0.10 ^b^	772.93 ± 7.90 ^g^	<LOD	<LOD	1.53 ± 0.07 ^c^	0.30 ± 0.01 ^c^	<LOD	<LOD	<LOD	–	–
SE-CȘ-O1	8.57 ± 1.16 ^b^	778.22 ± 10.84 ^e^	<LOD	<LOD	1.63 ± 0.10 ^b^	0.37 ± 0.08 ^a^	<LOD	<LOD	<LOD	–	–
SE-CȘ-O2	8.10 ± 0.10 ^b^	766.72 ± 4.06 ^i^	<LOD	<LOD	1.59 ± 0.10 ^c^	0.36 ± 0.04 ^a^	<LOD	<LOD	<LOD	–	–
SE-CȘ-O3	8.20 ± 0.23 ^b^	776.00 ± 9.09 ^f^	<LOD	<LOD	1.61 ± 0.10 ^b^	0.34 ± 0.01 ^a^	<LOD	<LOD	<LOD	–	–
SE-TNN-O1	7.76 ± 0.07 ^c^	771.29 ± 14.22 ^h^	<LOD	<LOD	1.64 ± 0.09 ^b^	0.32 ± 0.02 ^a^	<LOD	<LOD	<LOD	–	–
SE-UPCFBM-O1	8.38 ± 0.08 ^b^	780.08 ± 4.28 ^d^	<LOD	<LOD	1.67 ± 0.16 ^b^	0.32 ± 0.04 ^a^	<LOD	<LOD	<LOD	–	–
Zone III includes the control area											
SE-T-O1-O10	2.68 ± 0.02 ^d^	599.99 ± 7.34 ^m^	<LOD	<LOD	0.17 ± 0.04 ^e^	<LOD	<LOD	<LOD	<LOD	–	–
Kruskal–Wallis H *p*-value (Polluted Area vs. Control Area)	2.58 0.108	2.84 0.124	–	–	17.28 0.001	17.29 0.001	–	–	–	–	–
Spearman’s r (r^1^) *p*-value (*p*^1^)	0.464 0.111	0.463 0.111	–	–	0.460 0.133	0.492 0.104	–	–	–	–	–
Spearman’s r (r^2^) *p*-value (*p*^2^)	0.472 0.103	0.472 0.109	–	–	0.907 0.001	0.907 0.001	–	–	–	–	–
Comparative Analysis of Heavy Metal Levels in Horse Serum Samples, Including Samples from Control Areas											
Non-polluted area											
Oztas et al. (2025) [48] µg/mL	–	–	1.29	0.27	–	–	0.26	–	–	–	–
Brummer-Holder et al. (2022) [30] mg/L	–	–	BLD	BLD	BLD	–	BLD	–	–	–	–
Polluted area											
Maia et al. (2006) [49] µg/mL	–	–	0.021	0.010	0.003	–	–	–	–	–	–

SE = Serum; TMBTP = Tăuții-Măgherăuș/Bozânta Mare tailings ponds; RS = Recea/Săsar; TMN = Tăuții-Măgherăuș/Nistru; TMH = Tăuții-Măgherăuș/Herja; CI = Cicârlău/Ilba; CȘ = Cavnic/Șuior; UPCFBM = UP Central Flotation/Bozânta Mare; Tîrlișua = control area. O1–O10 = individual owners. Zone I (n = 9), Zone II (n = 27), and Zone III (n = 30) correspond to tailings, mining, and control areas, respectively. Kruskal–Wallis H = non-parametric test for multiple group comparison; Spearman’s ρ = rank correlation coefficient (where ρ represents the Spearman rank correlation coefficient). Different lowercase letters (a, b, c) indicate statistically significant differences (*p* < 0.05, one-way ANOVA followed by Tukey’s HSD test). BLD = Below Limit of Detection (Lowercase letters placed above the bars/values denote statistically significant differences between groups, as determined by one-way ANOVA with Tukey’s HSD post hoc test (*p* < 0.05). Negative ρ values (where ρ represents the Spearman rank correlation coefficient) indicate decreasing metal concentrations from contaminated to control zones, reflecting inverse correlations with pollution gradients. Limits of quantification (LoQ): Pb—0.231 µg/L; Cd—0.069 µg/L; Co—0.136 µg/L; As—0.743 µg/L; Hg—0.1379 µg/L. These define the lowest quantifiable concentrations with acceptable analytical precision.

**Table 4:** Concentrations of Heavy Metals (Cu, Zn, Pb, Cd, Ni, Co, As, Cr, Hg) in Equine Synovial Fluid Samples from Distinct Environmental Exposure Zones: Comparative Data from Former Mining Sites, Tailings Areas, and Control Regions (µg/L).

Sample Codes/Heavy Metals	Cu	Zn	Pb	Cd	Ni	Co	As	Cr	Hg	Spearman’s r	*p*-Value
Zone I encompass the areas of the former tailing’s ponds at Bozânta Mare, Săsar, and Nistru											
SF-TMBTP-O1	8.25 ± 2.85 ^a^	336.03 ± 15.78	<LOD	<LOD	<LOD	<LOD	<LOD	<LOD	<LOD	–	–
SF-RS-O1	9.05 ± 3.22 ^a^	307.29 ± 12.16	<LOD	<LOD	<LOD	<LOD	<LOD	<LOD	<LOD	–	–
SF-TMN-O1	5.54 ± 0.97 ^b^	271.41 ± 21.86	<LOD	<LOD	<LOD	<LOD	<LOD	<LOD	<LOD	–	–
Zone II includes the areas of the former mines: Herja, Ilba, Șuior, Nistru, and UP Central Flotation											
SF-TMH-O1	8.23 ± 1.08 ^a^	141.87 ± 14.97	<LOD	<LOD	<LOD	<LOD	<LOD	<LOD	<LOD	–	–
SF-TMH-O2	5.59 ± 1.05 ^b^	394.63 ± 7.40	<LOD	<LOD	<LOD	<LOD	<LOD	<LOD	<LOD	–	–
SF-CI-O1	4.42 ± 2.47 ^c^	222.78 ± 20.87	<LOD	<LOD	<LOD	<LOD	<LOD	<LOD	<LOD	–	–
SF-CI-O2	9.07 ± 1.71 ^a^	392.23 ± 24.07	<LOD	<LOD	<LOD	<LOD	<LOD	<LOD	<LOD	–	–
SF-CȘ-O1	4.57 ± 0.96 ^b^	108.87 ± 13.06	<LOD	<LOD	<LOD	<LOD	<LOD	<LOD	<LOD	–	–
SF-CȘ-O2	6.51 ± 2.00 ^b^	288.56 ± 33.51	<LOD	<LOD	<LOD	<LOD	<LOD	<LOD	<LOD	–	–
SF-CȘ-O3	3.37 ± 1.97 ^d^	296.30 ± 24.85	<LOD	<LOD	<LOD	<LOD	<LOD	<LOD	<LOD	–	–
SF-TNN-O1	4.70 ± 2.82 ^c^	217.92 ± 21.87	<LOD	<LOD	<LOD	<LOD	<LOD	<LOD	<LOD	–	–
SF-UPCFBM-O1	3.49 ± 1.32 ^d^	238.76 ± 25.25	<LOD	<LOD	<LOD	<LOD	<LOD	<LOD	<LOD	–	–
Zone III includes the control area											
SF-T-O1-O10	5.09 ± 1.36 ^b^	127.50 ± 25.25	<LOD	<LOD	<LOD	<LOD	<LOD	<LOD	<LOD	–	–
Kruskal–Wallis H *p*-value (Polluted Area vs. Control Area)	5.33 0.021	1.79 0.181	–	–	–	–	–	–	–	–	–
Spearman’s r (r^1^) *p*-value (*p*^1^)	0.460 0.133	0.386 0.193	–	–	–	–	–	–	–	–	–
Spearman’s r (r^2^) *p*-value (*p*^2^)	0.492 0.104	−0.014 0.965	–	–	–	–	–	–	–	–	–

SF = Synovial Fluid; TMBTP = Tăuții-Măgherăuș/Bozânta Mare tailings ponds; RS = Recea/Săsar; TMN = Tăuții-Măgherăuș/Nistru; TMH = Tăuții-Măgherăuș/Herja; CI = Cicârlău/Ilba; CȘ = Cavnic/Șuior; UPCFBM = UP Central Flotation/Bozânta Mare; Tîrlișua = control area. O1–O10 = individual owners. Zone I (n = 9), Zone II (n = 27), and Zone III (n = 30) correspond to tailings, mining, and control areas, respectively. Kruskal–Wallis H = non-parametric test for multiple group comparison; Spearman’s ρ = rank correlation coefficient (where ρ represents the Spearman rank correlation coefficient). Different lowercase letters (a, b, c) indicate statistically significant differences (*p* < 0.05, one-way ANOVA followed by Tukey’s HSD test) Lowercase letters placed above the bars/values denote statistically significant differences between groups, as determined by one-way ANOVA with Tukey’s HSD post hoc test (*p* < 0.05). <LOD = Below Limit of Detection. Negative ρ values indicate decreasing metal concentrations from contaminated to control zones, reflecting inverse correlations with pollution gradients (where ρ represents the Spearman rank correlation coefficient). Limits of quantification (LoQ): Pb—0.231 µg/L; Cd—0.069 µg/L; Co—0.136 µg/L; As—0.743 µg/L; Hg—0.1379 µg/L. These define the lowest quantifiable concentrations with acceptable analytical precision. Based on an extensive review of the scientific literature, no studies have been identified that report concentrations of heavy metals (such as Cu, Zn, Pb, Cd, Ni, Co, As, Cr, Hg) in equine synovial fluid. Most existing research focuses on analyzing synovial fluid for inflammatory markers, proteins, and other biochemical components, rather than heavy metals.

Zone II showed the highest metal concentrations in vegetation. Cu ranged from 1.6 to 12.5 mg/kg in grass and 1.6–11.5 mg/kg in hay; Zn from 113 to 167 mg/kg in grass and 102–151 mg/kg in hay. Pb reached 3.48–7.91 mg/kg in grass and 2.92–7.21 mg/kg in hay, exceeding the 10 mg/kg feed limit. Cd ranged from 0.20 to 0.47 mg/kg in grass and 0.16–0.37 mg/kg in hay. Ni measured 0.79–1.84 mg/kg in grass and 0.61–1.44 mg/kg in hay. Co, As, Cr, and Hg were below detection limits. Vegetation from the control area showed the lowest concentrations: Cu 0.16–0.54 mg/kg (grass) and 0.26–0.34 mg/kg (hay); Zn 12.6–29.1 mg/kg (grass) and 20.2–23.6 mg/kg (hay); Pb 0.16–0.89 mg/kg (grass) and 0.16–0.42 mg/kg (hay); Cd 0.04–0.07 mg/kg. Ni, Co, As, Cr, and Hg were below detection limits. A clear contamination gradient was observed (Zone II > Zone I > Zone III). Exceedances of regulatory or background limits were recorded for Cu, Zn, Pb, and Cd in Zones I–II, while Zone III values were within acceptable limits. RSD values were below 10% for Cu and Zn, indicating good analytical precision, whereas Pb showed higher variability.

### 3.7. Heavy Metal Profiles in Equine Feed Concentrates (Corn) from Polluted and Control Areas

Corn-based concentrate samples were available and analyzed exclusively in Zone III (control area). In Zones I and II, no such samples were collected, as concentrates were not administered to horses during the study period; feeding relied entirely on forage. This distinction is relevant for data interpretation, ensuring that the reported values reflect background feed conditions rather than anthropogenic inputs.

Given the absence of specific regulatory thresholds for heavy metals in corn-based concentrates intended for equine feeding, the evaluation was conducted by reference to the maximum permissible levels for forage materials (grass and hay) established in Directive 2002/32/EC (Annex I). These limits—Lead (Pb) 10.0 mg/kg, Cadmium (Cd) 1.0 mg/kg, Mercury (Hg) 0.1 mg/kg, Arsenic (As) 2.0 mg/kg, and Fluorine (F) 30.0 mg/kg—served as comparative benchmarks.

In the control zone, metal concentrations in vegetation were low and within reference limits. Cu ranged from 1.77 to 1.82 mg/kg (mean 1.80 mg/kg) and Zn from 27.56 to 28.45 mg/kg (mean 28.05 mg/kg). Pb was detected only at trace levels (0.03–0.04 mg/kg), representing less than 1% of the permissible limit. Cd, As, and Hg were below detection limits (BLD). Ni (0.09–0.11 mg/kg) and Co (0.04–0.06 mg/kg) occurred at trace levels within reported background ranges, and Cr was low (0.39–0.42 mg/kg). The heavy metal profile of corn-based concentrates from Zone III showed background levels dominated by essential trace elements (Cu, Zn), with toxic metals (Pb, Cd, As, Hg) either undetected or present at negligible concentrations. These results confirm the absence of contamination in control feed materials and provide a baseline reference. As concentrates were not used in Zones I and II, direct inter-zone comparisons were not possible.

### 3.8. Heavy Metal Profiles in Soil Samples from Polluted and Control Areas

The spatial distribution of heavy metals in soils showed marked contrasts among the three investigated zones, with clear enrichment in mining-affected areas (Zones I and II) and background levels in the control area (Zone III). Concentrations were compared with national soil quality standards defined by Order 756/1997 [50], (Table S24), and descriptive statistics are summarized in Table S25.

Soils exhibited systematic exceedances of legal thresholds. Cu ranged from 56.68 to 95.37 mg/kg (mean 74.70), more than three times the permissible limit (20 mg/kg). Zn showed the highest enrichment (288.06–438.13 mg/kg; mean 357.41), exceeding the limit of 100 mg/kg by up to fourfold. Pb values (25.13–36.16 mg/kg; mean 30.33) were above the 20 mg/kg limit, while Cd (2.74–3.54 mg/kg; mean 3.13) exceeded the 1 mg/kg threshold in all samples. Ni (22.96–28.91 mg/kg) and Co (18.61–21.03 mg/kg) were above natural background levels, confirming polymetallic enrichment.

The highest contamination occurred here. Cu reached 277.17 mg/kg (mean 156.71), surpassing both the legal limit (20 mg/kg) and the intervention threshold for susceptible soils (200 mg/kg). Zn ranged between 191.08 and 709.31 mg/kg (mean 416.86), four to seven times higher than the 100 mg/kg limit. Pb (13.59–42.26 mg/kg; mean 30.68) frequently exceeded permissible levels, while Cd (1.93–3.58 mg/kg; mean 2.71) consistently surpassed the 1 mg/kg threshold. Ni (19.73–23.01 mg/kg) and Co (17.14–22.22 mg/kg) were also elevated, reflecting the polymetallic nature of the deposits.

Soils exhibited background concentrations well below regulatory thresholds. Cu ranged from 8.95 to 9.45 mg/kg (mean 9.20), Zn 42.05–65.12 mg/kg (mean 50.65), Pb 12.50–13.26 mg/kg (mean 12.87), and Cd 0.41–0.74 mg/kg (mean 0.57). Ni (11.84–12.56 mg/kg) and Co (8.95–9.08 mg/kg) were consistent with geogenic values. As, Cr, and Hg were below detection limits across all sites.

A distinct contamination gradient was observed (Zone II > Zone I > Zone III). Cu, Zn, Pb, and Cd exceeded legal or alert thresholds in Zones I–II, while the control soils displayed natural background levels. These results confirm the strong influence of historical mining on soil metal accumulation and the suitability of Zone III as a geochemical reference.

### 3.9. Cumulative Pollution Index (CPI) Across Environmental and Biological Matrices

**Figure 1:**
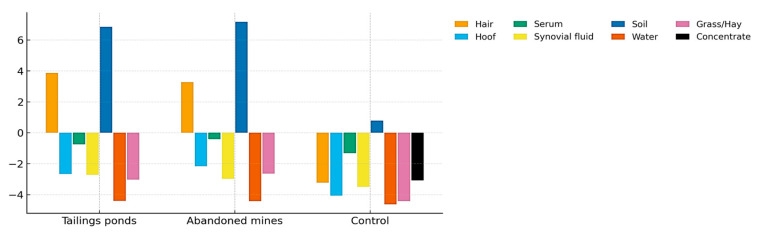
Cumulative Pollution Index (CPI) by Zone and Sample Matrix.

**Figure 2:**
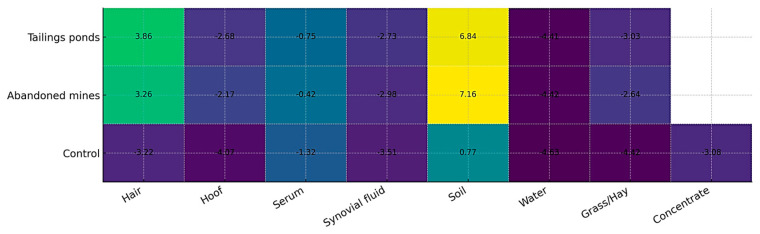
Composite Contamination (CPI) Heatmap: Tailings Ponds, Abandoned Mines, Control.

Figure 1 and Figure 2 illustrate the integrated distribution of heavy metals across environmental (soil, water, forage) and biological (hair, hoof, serum, synovial fluid) matrices within the three study zones. A clear gradient was observed, with markedly higher values in contaminated sites (tailings ponds and abandoned mines) compared to the control area.

Soil exhibited the highest enrichment, confirming its role as the primary sink and source of metal dispersion. Water samples from contaminated areas displayed negative deviations relative to the control, reflecting impaired quality and potential leaching from tailings deposits. Forage (grass and hay) also showed negative responses, indicating reduced nutritional quality and acting as a transfer pathway of contaminants into animal tissues.

Figure 1 presents the Cumulative Pollution Index (CPI) values for each matrix across the three study zones. CPI values represent the summed ratios between metal concentrations measured in polluted areas and the corresponding reference concentrations from the control zone $\left(CPI=\sum (C_{i}/C_{ref})\right)$. Values above zero indicate enrichment relative to control, whereas negative values indicate lower concentrations than background levels. Higher CPI values therefore reflect stronger contamination and bioaccumulation potential, whereas values near zero suggest minimal differentiation from baseline conditions. Soil exhibited the highest CPI, confirming its role as the primary reservoir of metals, whereas hair and hoof showed moderate positive values, reflecting long-term exposure integration. Conversely, serum and synovial fluid showed mostly negative or near-zero values, indicating limited systemic accumulation and short-term physiological response.

Among biological samples, hair and hoof presented distinct increases in contaminated zones, demonstrating their reliability as non-invasive biomarkers of long-term exposure. Conversely, serum and synovial fluid exhibited lower or negative values, particularly in mining-affected areas, suggesting short-term systemic responses rather than direct accumulation.

Overall, Figure 1 and Figure 2 reveal a consistent contamination continuum: metals accumulate primarily in soil, are partially transferred through water and vegetation, and ultimately reach biological compartments. Hair and hoof effectively integrate chronic exposure, whereas serum and synovial fluid capture transient physiological alterations, together providing a comprehensive overview of environmental–biological interactions in mining-impacted ecosystems.

Figure 2 displays a heatmap of the Cumulative Pollution Index (CPI) across environmental (soil, water, grass/hay, concentrate) and biological (hair, hoof, serum, synovial fluid) matrices in the three study zones. Each square represents the CPI value calculated as the ratio between metal concentrations in contaminated zones and corresponding control values. The numbers inside the cells indicate the CPI values for each matrix–zone combination. Color intensity reflects relative contamination levels, with lighter/yellow shades representing higher CPI values (greater contamination and bioaccumulation potential) and darker/purple shades representing lower or background levels. Values close to zero indicate no substantial difference from control, whereas values above 1 reflect enrichment above baseline.

### 3.10. Integration of Environmental and Biological Matrices

Integrated analysis demonstrated a clear correspondence between environmental contamination and biological accumulation (Tables S26–S28). Water, soil, and forage represented the main reservoirs and exposure pathways, with frequent exceedances of EU limits for Cu, Zn, Pb, and Cd, while control-area concentrates remained within acceptable values. Among biological matrices, hair and hoof showed the highest and most consistent metal levels, indicating chronic exposure patterns. Hair exhibited multi-element enrichment (As, Cu, Cr, Pb, Zn, Ni, Cd), whereas hoof showed comparable trends, particularly for Cu, Zn, Pb, Ni, and Co. Serum reflected short-term exposure responses (notably Ni and Co), and synovial fluid showed weak or inconsistent detection. A contamination gradient was evident across matrices: soil > water > forage > hair > hoof > serum > synovial fluid, indicating progressive transfer from environmental compartments to equine tissues.

## 4. Discussion

The results demonstrate, for the first time in this region, a consistent multi-matrix contamination gradient (abandoned mines > tailing ponds > control), indicating a strong link between environmental contamination and biological accumulation. The alignment between metal levels in soil, water, forage, and keratinized tissues supports the trophic transfer of heavy metals within mining-impacted ecosystems. Hair and hoof emerged as reliable non-invasive biomarkers of long-term exposure. Both matrices displayed comparable metal patterns, with no significant differences between mane and tail hair or between hoof wall and sole, indicating uniform accumulation across sampling sites. These findings support the analytical interchangeability of hair types and hoof regions and reinforce their applicability in field-based biomonitoring.

In contrast, copper concentrations were consistently higher in the hoof wall than in the sole across all exposure zones. This pattern, consistent with previous findings (e.g., Tocci et al., 2017) [27], likely reflects structural and functional differences between tissues. The hoof wall, being more exposed and characterized by higher keratin turnover, facilitates greater metal accumulation, whereas the more protected and less vascularized sole retains lower levels.

Tail hair data remain limited, indicating the need for further validation of its use as a biomarker of chronic exposure. Serum and synovial fluid reflected short-term responses, with only Ni and Co showing significant increases in polluted areas, while most toxic metals were near or below detection limits. This supports the concept that circulating metals are rapidly transferred to keratinized tissues, reinforcing the superior diagnostic value of hair and hoof for long-term exposure monitoring. Environmental matrices confirmed the persistence of mining-related contamination: soil and water from impacted zones exceeded legal thresholds for Cu, Zn, Pb, Cd, and Ni, while forage showed corresponding enrichment and active transfer into the trophic chain. In contrast, corn-based concentrates from the control area met regulatory standards, validating the baseline reference zone.

**Figure 3:**
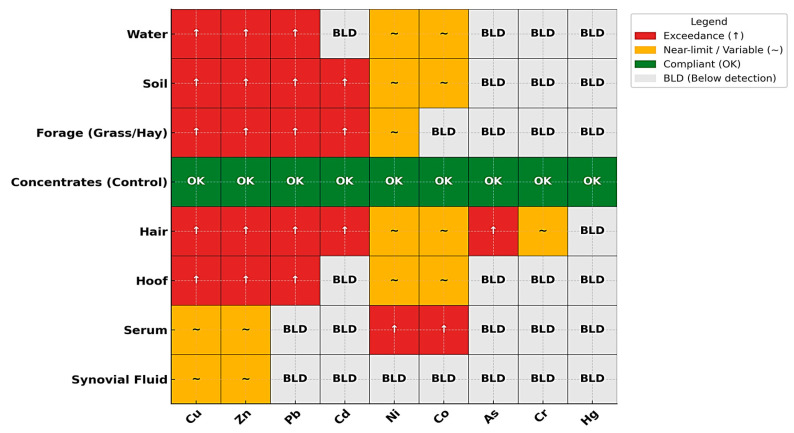
Compliance status and exceedances of heavy metals across environmental and biological matrices in horses.

Collectively, these results provide new evidence that integrating environmental (soil, water, vegetation) and biological (hair, hoof, serum) matrices yields a coherent picture of contamination pathways and biological accumulation in equine populations. The study establishes a transferable framework for multi-matrix biomonitoring in mining-impacted areas, with direct applications to environmental health surveillance and food safety management. Figure 3 illustrates the compliance status of heavy metals across environmental and biological matrices. Frequent exceedances in water, soil, and forage identify these as the principal reservoirs and transfer pathways of contamination, particularly for Cu, Zn, Pb, Ni, and Co.

Conversely, the control concentrates display full compliance, confirming their role as a reliable baseline reference. Biological matrices reflect these environmental patterns with high consistency. Hair and hoof exhibit multi-metal enrichments, confirming their reliability as indicators of chronic exposure. Serum shows selective increases, mainly for Ni and Co, corresponding to short-term systemic responses, whereas synovial fluid remains largely below detection thresholds. Near-limit values denote transitional exposure levels and highlight the sensitivity of biological responses to environmental metal loads.

**Figure 4:**
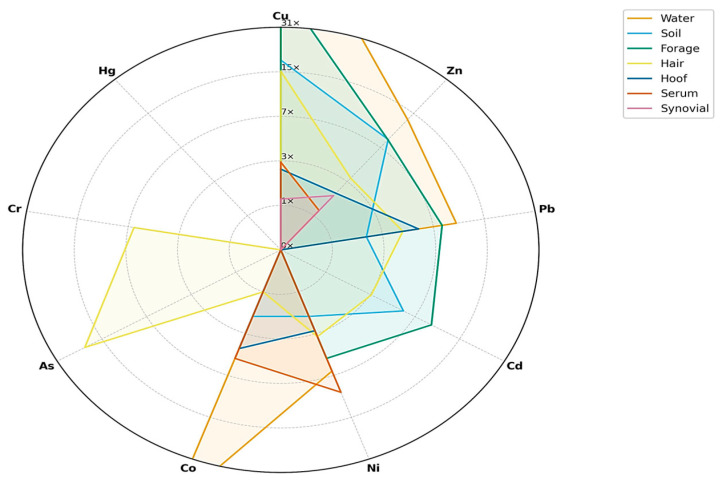
Comparative radar plot of heavy metal burdens across environmental and biological matrices in horses.

Figure 4 summarizes the relative distribution of heavy metals across environmental and biological matrices in contaminated zones using a radar chart. Each axis represents a single metal, and the plotted radial lines indicate normalized concentrations relative to control values (ratio = contaminated/control). Larger distances from the center reflect higher relative burdens. The different colored polygons represent sample matrices (soil, water, forage, hair, hoof, serum and synovial fluid), illustrating how metals distribute differently across compartments. The expanded areas for soil, water, hair and hoof demonstrate their higher capacity to accumulate metals, whereas serum and synovial fluid show shorter radii, indicating lower levels and transient systemic presence. The purpose of this plot is to visually compare the exposure profiles across matrices and identify which compartments best reflect contamination intensity.

**Figure 5:**
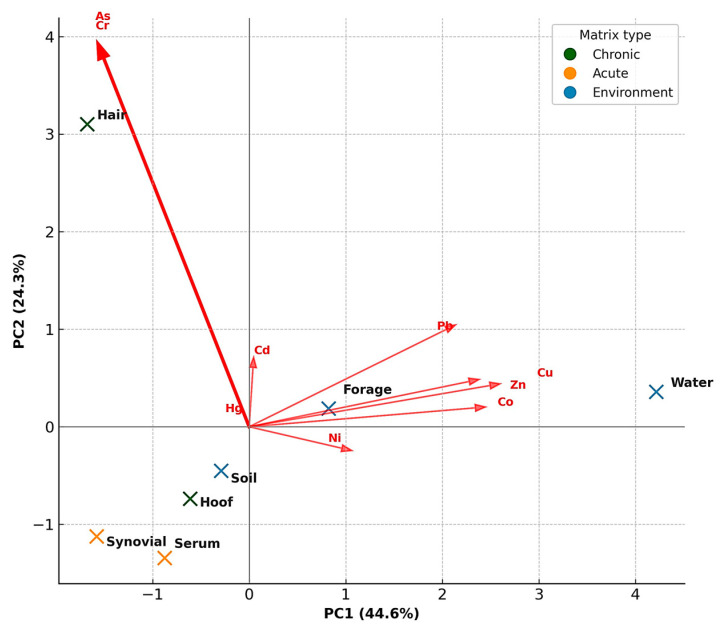
PCA biplot of heavy metal distribution across environmental and biological matrices in horses.

Figure 5 summarizes the multivariate relationships between heavy metals and sample matrices.

The PCA analysis distinguished three functional matrix clusters based on heavy metal profiles: (i) environmental (water, soil, forage), (ii) chronic biological (hair, hoof), and (iii) acute biological (serum, synovial fluid). Water showed the strongest association with Cu, Zn, Pb, Ni, and Co, indicating its key role in metal transport. Hair grouped with As and Cr, reflecting multi-element integration. Soil and hoof clustered closely, indicating similar long-term accumulation behavior. Serum and synovial fluid separated along the acute axis, capturing short-term variation. Overall, PCA demonstrates a clear gradient from environmental sources to biological uptake.

**Figure 6:**
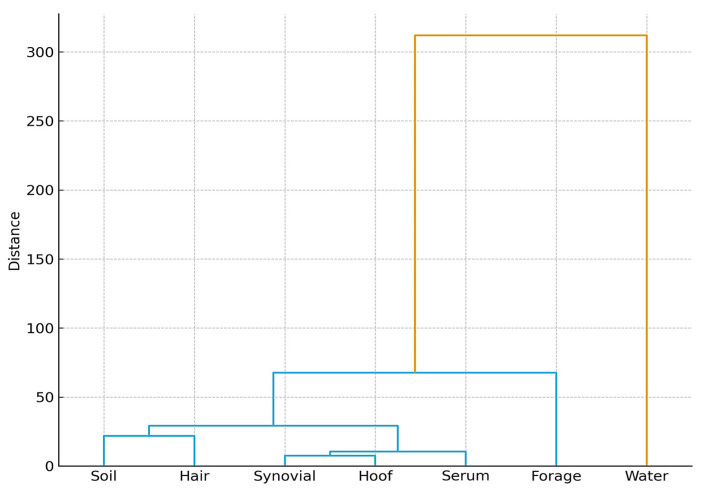
Hierarchical cluster analysis of environmental and biological matrices in relation to heavy metal burdens.

Hierarchical Cluster Analysis (HCA) grouped the matrices into distinct clusters based on their heavy metal profiles, showing strong similarity between soil, hoof, synovial fluid, and hair, whereas serum, forage, and water formed separate branches reflecting different accumulation behaviors (Figure 6).

## 5. Conclusions

The study revealed a clear contamination gradient across mining-affected zones, confirming a strong soil–plant–animal transfer of heavy metals. Hair and hoof proved to be reliable, non-invasive biomarkers of chronic exposure, showing consistent metal profiles and no significant differences between sample types. Serum reflected short-term exposure, while synovial fluid had limited diagnostic value. Soil and water were the main contamination sources, and forage acted as the dietary transfer pathway. Overall, horses represent effective sentinels for assessing heavy metal pollution within mining ecosystems.

## Figures and Tables

**Table 1:** Heavy Metal Concentrations (Cu, Zn, Pb, Cd, Ni, Co, As, Cr, Hg) in Equine Mane and Tail Hair Samples from Distinct Environmental Exposure Zones: Comparative Data from Former Mining, Tailings, and Control Areas (mg/kg FM).

Sample Codes/Heavy Metals	Cu	Zn	Pb	Cd	Ni	Co	As	Cr	Hg	Spearman’s r	*p*-Value
Zone, I encompass the areas of the former tailing’s ponds at Bozânta Mare, Săsar, and Nistru											
HA-M-TMBTP-O1	12.50 ± 1.32 ^c^	148.47 ± 2.50 ^e^	4.22 ± 0.14 ^e^	1.18 ± 0.29 ^b^	0.57 ± 0.12 ^b^	0.47 ± 0.03 ^c^	1.07 ± 0.14 ^e^	0.82 ± 0.02 ^bc^	BLD	−0.42	0.24
HA-T-TMBTP-O1	11.83 ± 1.30 ^c^	139.52 ± 0.90 ^f^	4.17 ± 0.09 ^e^	0.81 ± 0.25 ^d^	0.42 ± 0.10 ^d^	0.40 ± 0.02 ^d^	1.89 ± 0.02 ^a^	0.92 ± 0.01 ^a^	BLD	−0.38	0.30
HA-M-RS-O1	11.84 ± 3.62 ^c^	155.22 ± 2.80 ^e^	5.20 ± 0.09 ^d^	1.01 ± 0.41 ^c^	0.50 ± 0.16 ^c^	0.54 ± 0.02 ^c^	1.62 ± 0.02 ^b^	0.88 ± 0.01^b^	BLD	−0.35	0.33
HA-T-RS-O1	10.18 ± 0.36 ^c^	152.25 ± 4.54 ^e^	5.08 ± 0.07 ^d^	1.07 ± 0.49 ^b^	0.53 ± 0.20 ^b^	0.48 ± 0.02 ^c^	1.37 ± 0.03 ^d^	0.96 ± 0.01 ^a^	BLD	−0.37	0.31
HA-M-TMN-O1	15.08 ± 0.16 ^b^	170.38 ± 2.05 ^c^	6.11 ± 0.07 ^b^	0.91 ± 0.37 ^d^	0.46 ± 0.15 ^d^	0.60 ± 0.04 ^b^	0.74 ± 0.01 ^g^	0.88 ± 0.01 ^b^	BLD	−0.40	0.28
HA-T-TMN-O1	13.09 ± 0.33 ^c^	161.45 ± 0.91 ^d^	5.92 ± 0.04 ^b^	0.83 ± 0.17 ^d^	0.44 ± 0.07 ^d^	0.60 ± 0.02 ^b^	0.73 ± 0.03 ^g^	0.96 ± 0.01 ^a^	BLD	−0.39	0.29
Zone, II includes the areas of the former mines: Herja, Ilba, Șuior, Nistru, and UP Central Flotation											
HA-M-TMH-O1	18.91 ± 0.57 ^a^	186.60 ± 1.29 ^a^	7.40 ± 0.08 ^a^	0.94 ± 0.16 ^c^	0.48 ± 0.06 ^d^	0.52 ± 0.01 ^c^	0.60 ± 0.03 ^g^	0.61 ± 0.01 ^e^	BLD	−0.45	0.22
HA-T-TMH-O1	16.74 ± 0.42 ^a^	177.39 ± 0.97 ^b^	7.22 ± 0.03 ^a^	0.77 ± 0.12 ^d^	0.36 ± 0.05 ^d^	0.67 ± 0.02 ^a^	1.75 ± 0.04 ^b^	0.66 ± 0.01 ^e^	BLD	−0.43	0.25
HA-M-TMH-O2	17.45 ± 0.20 ^a^	183.65 ± 1.26 ^a^	7.58 ± 0.07 ^a^	0.98 ± 0.30 ^c^	0.49 ± 0.12 ^d^	0.58 ± 0.07 ^b^	1.43 ± 0.01 ^c^	0.56 ± 0.03 ^f^	BLD	−0.41	0.27
HA-T-TMH-O2	15.24 ± 0.46 ^b^	171.55 ± 1.01 ^c^	7.09 ± 0.05 ^a^	0.88 ± 0.29 ^d^	0.45 ± 0.12 ^d^	0.19 ± 0.07 ^f^	1.57 ± 0.02 ^c^	0.56 ± 0.01 ^f^	BLD	−0.44	0.24
HA-M-CI-O1	17.21 ± 1.42 ^a^	180.05 ± 1.52 ^a^	6.58 ± 0.07 ^b^	0.78 ± 0.29 ^d^	0.35 ± 0.02 ^e^	0.18 ± 0.05 ^f^	0.54 ± 0.04 ^h^	0.91 ± 0.01 ^a^	BLD	−0.46	0.21
HA-T-CI-O1	15.86 ± 0.13 ^b^	170.28 ± 0.88 ^c^	6.42 ± 0.03 ^b^	1.41 ± 0.09 ^a^	0.66 ± 0.03 ^a^	0.13 ± 0.02 ^g^	1.95 ± 0.02 ^a^	0.71 ± 0.01 ^d^	BLD	−0.42	0.26
HA-M-CI-O2	17.55 ± 0.19 ^a^	183.59 ± 1.40 ^a^	6.79 ± 0.03 ^a^	0.86 ± 0.29 ^d^	0.45 ± 0.12 ^d^	0.16 ± 0.05 ^f^	1.75 ± 0.05 ^b^	0.75 ± 0.01 ^d^	BLD	−0.44	0.24
HA-T-CI-O2	16.65 ± 0.20 ^b^	175.49 ± 1.20 ^b^	6.58 ± 0.03 ^b^	0.85 ± 0.20 ^d^	0.44 ± 0.08 ^d^	0.09 ± 0.07 ^g^	0.80 ± 0.02 ^f^	0.57 ± 0.01 ^f^	BLD	−0.43	0.25
HA-M-CȘ-O1	14.81 ± 0.14 ^b^	166.74 ± 1.63 ^c^	6.01 ± 0.04 ^b^	0.90 ± 0.46 ^c^	0.46 ± 0.18 ^d^	0.16 ± 0.02 ^f^	0.77 ± 0.03 ^g^	1.01 ± 0.01 ^a^	BLD	−0.41	0.27
HA-T-CȘ-O1	14.05 ± 0.10 ^b^	159.31 ± 0.91 ^d^	5.85 ± 0.04 ^b^	1.00 ± 0.18	0.50 ± 0.07 ^c^	0.13 ± 0.03 ^g^	0.79 ± 0.04 ^g^	0.85 ± 0.01 ^bc^	BLD	−0.54	0.36
HA-M-CȘ-O2	15.71 ± 0.31 ^b^	117.20 ± 1.28 ^c^	6.21 ± 0.07 ^b^	1.07 ± 0.40 ^b^	0.53 ± 0.16 ^b^	BLD	0.96 ± 0.03 ^f^	0.77 ± 0.01 ^d^	BLD	−0.45	0.22
HA-T-CȘ-O2	14.66 ± 0.12 ^b^	163.32 ± 1.03 ^d^	6.02 ± 0.04 ^b^	1.37 ± 0.08 ^b^	0.65 ± 0.03 ^a^	BLD	1.29 ± 0.04 ^d^	0.65 ± 0.01 ^e^	BLD	−0.43	0.24
HA-M-CȘ-O3	16.33 ± 0.09 ^b^	176.41 ± 0.76 ^b^	6.41 ± 0.04 ^b^	1.04 ± 0.42 ^a^	0.51 ± 0.17 ^c^	0.13 ± 0.03 ^g^	1.13 ± 0.01 ^f^	0.74 ± 0.01 ^d^	BLD	−0.42	0.25
HA-T-CȘ-O3	15.57 ± 0.17 ^b^	169.42 ± 1.05 ^c^	6.21 ± 0.04 ^b^	0.69 ± 0.14 ^d^	0.38 ± 0.06 ^e^	0.16 ± 0.05	0.94 ± 0.01 ^f^	0.79 ± 0.01 ^d^	BLD	−0.44	0.24
HA-M-TNN-O1	15.28 ± 3.52 ^b^	181.83 ± 1.39 ^a^	7.19 ± 0.07 ^a^	0.83 ± 0.06 ^d^	0.50 ± 0.12 ^c^	BLD	1.39 ± 0.03 ^d^	0.82 ± 0.04 ^bc^	BLD	−0.46	0.21
HA-T-TNN-O1	12.38 ± 0.15 ^c^	171.57 ± 1.06 ^c^	6.97 ± 0.04 ^b^	0.89 ± 0.13 ^c^	0.46 ± 0.05 ^d^	BLD	0.62 ± 0.13 ^g^	0.59 ± 0.01 ^f^	BLD	−0.40	0.28
HA-M-UPCFBM-O1	14.64 ± 0.10 ^b^	167.40 ± 1.59 ^c^	5.85 ± 0.04 ^b^	0.84 ± 0.40 ^d^	0.44 ± 0.16 ^d^	BLD	0.96 ± 0.13 ^f^	0.63 ± 0.05 ^e^	BLD	−0.41	0.27
HA-T-UPCFBM-O1	14.25 ± 0.31 ^b^	158.62 ± 0.96 ^d^	5.69 ± 0.04 ^c^	1.15 ± 0.41 ^a^	0.56 ± 0.16 ^b^	BLD	1.03 ± 0.01 ^e^	0.70 ± 0.01 ^d^	BLD	−0.39	0.29
Zone III includes the control area											
HA-M-T-O1-O10	1.12 ± 0.07 ^d^	51.36 ± 1.09 ^g^	1.49 ± 0.04 ^f^	0.35 ± 0.13 ^e^	BLD ^f^	0.04 ±0.01	0.06 ± 0.01 ^i^	0.12 ± 0.01 ^g^	BLD	−0.64	0.07
HA-T-T-O1-O10	1.01 ± 0.06 ^d^	48.56 ± 1.40 ^g^	1.32 ± 0.04 ^f^	0.36 ± 0.12 ^e^	0.16 ± 0.01 ^f^	BLD	0.08 ± 0.01 ^i^	0.13 ± 0.01 ^g^	BLD	−0.62	0.08
Kruskal–Wallis H *p*-value (Polluted Area vs. Control Area)	5.33 0.021	5.33 0.021	5.34 0.023	4.57 0.038	5.79 0.016	1.35 0.245	5.78 0.046	8.18 0.068	–	–	–
Kruskal–Wallis H *p*-value (Sample Type (Mane vs. Tail)	2.14 0.144	2.45 0.118	0.38 0.538	0.053 0.817	0.56 0.455	0.213 0.642	0.38 0.538	0.26 0.608	–	–	–
Spearman’s r (r^1^) *p*-value (*p*^1^)	0.29 0.147	−0.028 0.893	−0.052 0.801	−0.23 0.254	−0.21 0.316	0.993 -	−0.378 0.057	−0.103 0.618	–	–	–
Spearman’s r (r^2^) *p*-value (*p*^2^)	0.46 0.018	−0.028 0.893	0.206 0.335	−0.46 0.017	0.12 0.562	0.996 -	−0.023 0.912	−0.462 0.017	–	–	–
Spearman’s r (r^3^) *p*-value (*p*^3^)	−0.09 0.652	0.462 0.018	0.25 0.650	−0.046 0.823	0.45 0.176	0.982 -	−0.032 0.875	−0.499 0.001	–	–	–
Comparative Analysis of Heavy Metal Levels in Horse Mane and Tail Hair, Including Samples from Control Areas											
Hair specimens harvested from the upper neck (mane) area of horses											
Wahl et al., 2022 mg/kg FM [44]	4.19–7.59	110–214	–	–	–	0.02–0.22	–	–	–	–	–
Namkoong et al., 2013 µg/g [45]	–	–	0.20–9.25	0.03–0.37	–	–	0.02–0.62	–	0.17–7.89	–	–
Kwiecié et., 2022 mg/kg [11]	6.10–11.99	153.56–185.79	0.578–0.813	0.011–0.015	–	–	–	–	–	–	–

HA = Hair; M = Mane; T = Tail; TMBTP = Tăuții-Măgherăuș/Bozânta Mare tailings ponds; RS = Recea/Săsar; TMN = Tăuții-Măgherăuș/Nistru; TMH = Tăuții-Măgherăuș/Herja; CI = Cicârlău/Ilba; CȘ = Cavnic/Șuior; TNN = Tăuții-Măgherăuș/Nistru; UPCFBM = UP Central Flotation/Bozânta Mare; Tîrlișua = control area. O1–O10 = individual owners. Zone I (n = 9), Zone II (n = 27), and Zone III (n = 30) correspond to tailings, mining, and control areas, respectively. FM = Fresh Matter. Different lowercase letters (a, b, c) indicate statistically significant differences (*p* < 0.05, one-way ANOVA followed by Tukey’s HSD test) (Lowercase letters placed above the bars/values denote statistically significant differences between groups, as determined by one-way ANOVA with Tukey’s HSD post hoc test (*p* < 0.05). BLD = Below Limit of Detection. Kruskal–Wallis H = non-parametric test for group comparison; Spearman’s *ρ* = rank correlation coefficient. Negative *ρ* values indicate decreasing metal concentrations from contaminated to control zones, reflecting inverse correlations with pollution gradients. The limits of quantification (LoQ) determined for the analyzed heavy metals were as follows: Pb—0.231 µg/L, Cd—0.069 µg/L, Co—0.136 µg/L, As—0.743 µg/L, and Hg—0.1379 µg/L. These values define the lowest concentration levels at which each element could be quantitatively determined with acceptable precision and accuracy under the applied analytical conditions.

## Data Availability

All data supporting the results of this study are included in the article and its Supplementary Materials.

## Supplementary Materials

The following supporting information can be downloaded at: https://www.mdpi.com/article/10.3390/toxics13121064/s1, Table S1. Comprehensive Inventory of Equine Hair Samples: Detailed Records of Mane and Tail Specimens, Including Sampling Location, Owner Identification, and Chronological Data; Table S2. Detailed Inventory of Equine Hoof Wall and Sole Samples, Including Site, Owner, and Collection Date; Table S3. Detailed Inventory of Equine Serum Samples, Including Site, Owner, and Collection Date; Table S4. Detailed Inventory of Equine Synovial Fluid Samples, Including Site, Owner, and Collection Date; Table S5. Detailed Inventory of Water Samples, Including Site, Owner, and Collection Date; Table S6. Detailed Inventory of Vegetation Samples (Grass and Hay), Including Site, Owner, and Collection Date; Table S7. Detailed Inventory of Equine Feed Samples (Concentrates), Including Site, Owner, and Collection Date; Table S8. Detailed Inventory of Soil Samples, Including Site, Owner, and Collection Date; Table S9. The operational program of the Milestone START D Microwave Digestion System, which regulates the parameters for sample disaggregation and digestion; Table S10. The instrumental settings (a) and data acquisition parameters (b) of the ICP–MS system, which define the operating conditions and analytical procedures for precise metal quantification; Table S11. Instrumental conditions for the determination of each element using ICP–MS technique; Table S12. Validation parameters of the analytical procedure for the determination of heavy metals (mane and tail hair samples); Table S13. Validation parameters of the analytical procedure for the determination of heavy metals (hoof samples); Table S14. Validation parameters of the analytical procedure for the determination of heavy metals (Caprine Blood and Synovial Fluid); Table S15. Validation parameters of the analytical procedure for the determination of heavy metals (water samples); Table S16. Validation parameters of the analytical procedure for determination of heavy metals (green grass); Table S17. Validation parameters of the analytical procedure for the determination of heavy metals (concentrate feed – maize-based samples); Table S18. Validation parameters of the analytical procedure for the determination of heavy metals (soil); Table S19. Limits of Heavy Metals in Water – For Human Consumption, Surface Water, and Animal Use (Including Horses); Table S20. Assessment of Heavy metals Contamination Across Three Zone Based on Legal Drinking Water Limits: A Descriptive Comparison; Table S21. Legal Limits for Heavy Metals in Grass and Hay (Feed Use) Table S22. Assessment of Heavy Metal Accumulation in Grass and Hay Samples Across Three Zones Based on Legal Feed and Food Safety Limits: A Descriptive Comparison; Table S23. Equine Feed Concentrates (Corn) from the Control Area: Descriptive Statistics and Compliance Benchmarking; Table S24. Legal Thresholds for Heavy Metals in Agricultural Soils (Topsoil 0–10 cm, Dry Weight Basis) According to Romanian Guidelines; Table S25. Assessment of Heavy Metal Accumulation in Soil Samples Across Three Zones Based on Legal Soil Quality Standards: A Descriptive Comparison; Table S26. Summary of Environmental and Biological Matrices for Heavy Metal Assessment in Horses; Table S27. Percentage Increase in Dominant Heavy Metals in Horses and Environmental Matrices (Polluted vs. Control Zones); Table S28. Environmental Sources, Dominant Metals, EU Legal Compliance, and Biomarker Relevance in Horses [51,52,53,54,55,56,57,58,59,60,61,62,63,64,65,66,67].

## Abbreviations

The following abbreviations are used in this manuscript:

As	Arsenic
Cd	Cadmium
Co	Cobalt
Cr	Chromium
Cu	Copper
Hg	Mercury
Ni	Nickel
Pb	Lead
Zn	Zinc
ICP–MS	Inductively Coupled Plasma–Mass Spectrometry
SF	Synovial Fluid
SE	Serum
HA	Hair
HF	Hoof
W	Water
G	Grass
H	Hay
CF	Concentrate Feed
S	Soil
BLD	Below Detection Limit
LoQ	Limit of Quantification
RSD	Relative Standard Deviation
CPI	Cumulative Pollution Index
