# Supplementary material for: Sentinel Equines in Anthropogenic Landscapes: Bioaccumulation of Heavy Metals and Hematological Biomarkers as Indicators of Environmental Contamination
Source: Toxics. 2025 Dec 9;13(12):1064. doi: 10.3390/toxics13121064 (PMC12737071; doi:10.3390/toxics13121064)
Supplement: Supplementary file 1 [file toxics-13-01064-s001.zip › toxics-3981505-supplementary.pdf]

Table S1. Comprehensive Inventory of Equine Hair Samples: Detailed Records of Mane and Tail Specimens, Including Sampling Location, Owner Identification, and Chronological Data

Sample Code	Hair Collection Site	Zone/The main sources of pollution	Owner	Collection Date	Observations
Zone I encompasses the areas of the former tailings ponds at Bozânta Mare, Săsar, and Nistru					
HA-M-TMBTP-O1	Mane	TMBTP	O1	March 12, 2025	Length: 10 cm Color: brown
HA-T-TMBTP-O1	Tail	TMBTP	O1	March 12, 2025	Length: 30 cm Color: black
HA-M-RS-O1	Mane	RS	O1	March 12, 2025	Length: 8 cm Color: brown
HA-T-RS-O1	Tail	RS	O1	March 12, 2025	Length: 22 cm Color: black
HA-M-TMN-O1	Mane	TMN	O1	March 12, 2025	Length: 9 cm Color: brown
HA-T-TMN-O1	Tail	TMN	O1	March 12, 2025	Length: 25 cm Color: black
Zone II includes the areas of the former mines: Herja, Ilba, Șuior, Nistru, and UP Central Flotation					
HA-M-TMH-O1	Mane	TMH	O1	March 12, 2025	Length: 9 cm Color: brown
HA-T-TMH-O1	Tail	TMH	O1	March 12, 2025	Length: 30 cm Color: brown
HA-M-TMH-O2	Mane	TMH	O2	March 12, 2025	Length: 9 cm Color: black
HA-T-TMH-O2	Tail	TMH	O2	March 12, 2025	Length: 30 cm Color: black
HA-M-CI-O1	Mane	CI	O1	March 7, 2025	Length: 10 cm Color: brown
HA-T-CI-O1	Tail	CI	O1	March 7, 2025	Length: 30 cm Color: brown
HA-M-CI-O2	Mane	CI	O2	March 7, 2025	Length: 10 cm Color: brown
HA-T-CI-O2	Tail	CI	O2	March 7, 2025	Length: 30 cm Color: brown
HA-M-CȘ-O1	Mane	CȘ	O1	March 7, 2025	Length: 5 cm Color: black
HA-T-CȘ-O1	Tail	CȘ	O1	March 7, 2025	Length: 30 cm Color: black
HA-M-CȘ-O2	Mane	CȘ	O2	March 7, 2025	Length: 15 cm Color: white
HA-T-CȘ-O2	Tail	CȘ	O2	March 7, 2025	Length: 30 cm

					Color: white
HA-M-CȘ-O3	Mane	CȘ	O3	March 7, 2025	Length: 12 cm<br>Color: black
HA-T-CȘ-O3	Tail	CȘ	O3	March 7, 2025	Length: 30 cm<br>Color: black
HA-M-TNN-O1	Mane	TNN	O1	March 7, 2025	Length: 10 cm<br>Color: brown
HA-T-TNN-O1	Tail	TNN	O1	March 7, 2025	Length: 30 cm<br>Color: brown
HA-M-UPCFBM-O1	Mane	UPCFBM	O1	March 7, 2025	Length: 11 cm<br>Color: black
HA-T-UPCFBM-O1	Tail	UPCFBM	O1	March 7, 2025	Length: 27 cm<br>Color: black
Zone III includes the control area					
HA-M-T-O1	Mane	Tîrlișua	O1	March 7, 2025	Length: 7 cm<br>Color: black
HA-T-T-O1	Tail	Tîrlișua	O1	March 7, 2025	Length: 25 cm<br>Color: black
HA-M-T-O2	Mane	Tîrlișua	O2	February 10-11, 2025	Length: 10 cm<br>Color: brown
HA-T-T-O2	Tail	Tîrlișua	O2	February 10-11, 2025	Length: 30 cm<br>Color: brown
HA-M-T-O3	Mane	Tîrlișua	O3	February 10-11, 2025	Length: 15 cm<br>Color: white
HA-T-T-O3	Tail	Tîrlișua	O3	February 10-11, 2025	Length: 30 cm<br>Color: white
HA-M-T-O4	Mane	Tîrlișua	O4	February 10-11, 2025	Length: 9 cm<br>Color: black
HA-T-T-O4	Tail	Tîrlișua	O4	February 10-11, 2025	Length: 27 cm<br>Color: black
HA-M-T-O5	Mane	Tîrlișua	O5	February 10-11, 2025	Length: 12 cm<br>Color: black
HA-T-T-O5	Tail	Tîrlișua	O5	February 10-11, 2025	Length: 30 cm<br>Color: black
HA-M-T-O6	Mane	Tîrlișua	O6	February 10-11, 2025	Length: 13 cm<br>Color: black
HA-T-T-O6	Tail	Tîrlișua	O6	February 10-11, 2025	Length: 30 cm<br>Color: black
HA-M-T-O7	Mane	Tîrlișua	O7	February 10-11, 2025	Length: 10 cm<br>Color: brown
HA-T-T-O7	Tail	Tîrlișua	O7	February 10-11, 2025	Length: 30 cm<br>Color: brown
HA-M-T-O8	Mane	Tîrlișua	O8	February 10-11, 2025	Length: 10 cm

					Color: black
HA-T-T-O8	Tail	Tîrlișua	O8	February 10-11, 2025	Length: 30 cm<br>Color: black
HA-M-T-O9	Mane	Tîrlișua	O9	February 10-11, 2025	Length: 10 cm<br>Color: black
HA-T-T-O9	Tail	Tîrlișua	O9	February 10-11, 2025	Length: 30 cm<br>Color: black
HA-M-T-O10	Mane	Tîrlișua	O10	February 10-11, 2025	Length: 10 cm<br>Color: brown
HA-T-T-O10	Tail	Tîrlișua	O10	February 10-11, 2025	Length: 30 cm<br>Color: brown

HA = Hair; M = Mane; T = Tail; TMBTP = Tăuții-Măgherăuș / Bozânta Mare tailings pods; RS = Recea / Săsar; TMN = Tăuții-Măgherăuș / Nistru; TMH = Tăuții-Măgherăuș / Herja; CI = Cicârlău / Ilba; CȘ = Cavnic / Șuior; Tăuții-Măgherăuș / Nistru = TNN; UPCFBM = UP Central Flotation / Bozânta Mare; Tîrlișua = T. O1 = Owner number 1; O2 = Owner number 2; O3 = Owner number 3; O4 = Owner number 4; O5 = Owner number 5; O6 = Owner number 6; O7 = Owner number 7; O8 = Owner number 8; O9 = Owner number 9; O10 = Owner number 10. Zone I n = 9 (hair samples and mane hair samples in common); Zone II n = 27 (hair samples and mane hair samples in common); Zone III n = 30 (hair samples and mane hair samples in common). Sample code format: HA–[M/T]–[Site Code]–[Owner ID].

Table S2. Detailed Inventory of Equine Hoof Wall and Sole Samples, Including Site, Owner, and Collection Date

**Sample Code**	**Hoof Collection Site**	**Zone/The main sources of pollution**	**Owner**	**Collection Date**	**Observations**
Zone I encompasses the areas of the former tailings ponds at Bozânta Mare, Săsar, and Nistru					
HF-W-TMBTP-O1	Wall	TMBTP	O1	March 12, 2025	Keratinized<br>Slightly soft
HF-S-TMBTP-O1	Sole	TMBTP	O1	March 12, 2025	Keratinized<br>Slightly smooth
HF-W-RS-O1	Wall	RS	O1	March 12, 2025	Keratinized<br>Slightly smooth
HF-S-RS-O1	Sole	RS	O1	March 12, 2025	Keratinized<br>Discolored
HF-W-TMN-O1	Wall	TMN	O1	March 12, 2025	Keratinized;<br>Slightly smooth
HF-S-TMN-O1	Sole	TMN	O1	March 12, 2025	Keratinized<br>Discolored
Zone II includes the areas of the former mines: Herja, Ilba, Șuior, Nistru, and UP Central Flotation					
HF-W-TMH-O1	Wall	TMH	O1	March 12, 2025	Keratinized;<br>Slightly smooth
HF-S-TMH-O1	Sole	TMH	O1	March 12, 2025	Keratinized<br>Matte surface
HF-W-TMH-O2	Wall	TMH	O2	March 12, 2025	Keratinized<br>Rough surface

HF-S-TMH-O2	Sole	TMH	O2	March 12, 2025	Keratinized<br>Slightly smooth
HF-W-CI-O1	Wall	CI	O1	March 7, 2025	Keratinized<br>Discolored
HF-S-CI-O1	Sole	CI	O1	March 7, 2025	Cracked<br>Slightly rough
HF-W-CI-O2	Wall	CI	O2	March 7, 2025	Porous; Matte<br>Signs of erosion
HF-S-CI-O2	Sole	CI	O2	March 7, 2025	Keratinized<br>Discolored
HF-W-CȘ-O1	Wall	CȘ	O1	March 7, 2025	Keratinized<br>Slightly smooth
HF-S-CȘ-O1	Sole	CȘ	O1	March 7, 2025	Keratinized<br>Slightly smooth
HF-W-CȘ-O2	Wall	CȘ	O2	March 7, 2025	Keratinized;<br>Slightly smooth
HF-S-CȘ-O2	Sole	CȘ	O2	March 7, 2025	Keratinized<br>Porous; Matte
HF-W-CȘ-O3	Wall	CȘ	O3	March 7, 2025	Keratinized<br>Discolored
HF-S-CȘ-O3	Sole	CȘ	O3	March 7, 2025	Keratinized<br>Discolored
HF-W-TNN-O1	Wall	TNN	O1	March 7, 2025	Keratinized<br>Slightly smooth
HF-S-TNN-O1	Sole	TNN	O1	March 7, 2025	Keratinized<br>Porous; Matte
HF-W-UPCFBM-O1	Wall	UPCFBM	O1	March 7, 2025	Keratinized<br>Slightly smooth
HF-S-UPCFBM-O1	Sole	UPCFBM	O1	March 7, 2025	Keratinized<br>Slightly smooth
Zone III includes the control area					
HF-W-T-O1	Wall	Tîrlișua	O1	March 7, 2025	Keratinized<br>Discolored
HF-S-T-O1	Sole	Tîrlișua	O1	March 7, 2025	Keratinized<br>Slightly smooth
HF-W-T-O2	Wall	Tîrlișua	O2	February 10-11, 2025	Keratinized<br>Discolored
HF-S-T-O2	Sole	Tîrlișua	O2	February 10-11, 2025	Keratinized<br>Discolored
HF-W-T-O3	Wall	Tîrlișua	O3	February 10-11, 2025	Keratinized<br>Slightly smooth
HF-S-T-O3	Sole	Tîrlișua	O3	February 10-11, 2025	Cracked<br>Slightly rough

HF-W-T-O4	Wall	Tîrlișua	O4	February 10-11, 2025	Keratinized Discolored
HF-S-T-O4	Sole	Tîrlișua	O4	February 10-11, 2025	Keratinized Discolored
HF-W-T-O5	Wall	Tîrlișua	O5	February 10-11, 2025	Keratinized Discolored
HF-S-T-O5	Sole	Tîrlișua	O5	February 10-11, 2025	Cracked Slightly rough
HF-W-T-O6	Wall	Tîrlișua	O6	February 10-11, 2025	Keratinized Discolored
HF-S-T-O6	Sole	Tîrlișua	O6	February 10-11, 2025	Keratinized Discolored
HF-W-T-O7	Wall	Tîrlișua	O7	February 10-11, 2025	Keratinized Discolored
HF-S-T-O7	Sole	Tîrlișua	O7	February 10-11, 2025	Cracked Slightly rough
HF-W-T-O8	Wall	Tîrlișua	O8	February 10-11, 2025	Keratinized Slightly smooth
HF-S-T-O8	Sole	Tîrlișua	O8	February 10-11, 2025	Cracked; Slightly rough
HF-W-T-O9	Wall	Tîrlișua	O9	February 10-11, 2025	Keratinized Slightly smooth
HF-S-T-O9	Sole	Tîrlișua	O9	February 10-11, 2025	Cracked; Slightly rough
HF-W-T-O10	Wall	Tîrlișua	O10	February 10-11, 2025	Keratinized Slightly smooth
HF-S-T-O10	Sole	Tîrlișua	O10	February 10-11, 2025	Keratinized Slightly smooth

HF = Hoof; W = Wall; S = Sole; TMBTP = Tăuții-Măgherăuș / Bozânta Mare tailings pods; RS = Recea / Săsar; TMN = Tăuții-Măgherăuș / Nistru; TMH = Tăuții-Măgherăuș / Herja; CI = Cicârlău / Ilba; CȘ = Cavnic / Șuior; Tăuții-Măgherăuș / Nistru = TNN; UPCFBM = UP Central Flotation / Bozânta Mare; Tîrlișua = T. O1 = Owner number 1; O2 = Owner number 2; O3 = Owner number 3; O4 = Owner number 4; O5 = Owner number 5; O6 = Owner number 6; O7 = Owner number 7; O8 = Owner number 8; O9 = Owner number 9; O10 = Owner number 10. Zone I n = 9 (wall samples and sole samples in common); Zone II n = 27 (wall samples and sole samples in common); Zone III n = 30 (wall samples and sole samples in common). Sample code format: HF–[W/S]–[Site Code]–[Owner ID].

Table S3. Detailed Inventory of Equine Serum Samples, Including Site, Owner, and Collection Date

Sample Code	Sample Type	Zone/The main sources of pollution	Owner	Collection Date	Observations
Zone I encompasses the areas of the former tailings ponds at Bozânta Mare, Săsar, and Nistru					
SE-TMBTP-O1	Serum	TMBTP	O1	October 12, 2025	Clear Slightly yellow
SE-RS-O1	Serum	RS	O1	October 12, 2025	Clear

					Slightly yellow
SE-TMN-O1	Serum	TMN	O1	October 12, 2025	Clear<br>Slightly yellow
Zone II includes the areas of the former mines: Herja, Ilba, Șuior, Nistru, and UP Central Flotation					
SE-TMH-O1	Serum	TMH	O1	October 12, 2025	Clear<br>Slightly yellow
SE-TMH-O2	Serum	TMH	O2	October 12, 2025	Clear<br>Slightly yellow
SE-CI-O1	Serum	CI	O1	October 7, 2025	Discolored<br>Slightly hemolyzed
SE-CI-O2	Serum	CI	O2	October 7, 2025	Clear<br>Slightly yellow
SE-CȘ-O1	Serum	CȘ	O1	October 7, 2025	Discolored<br>Slightly hemolyzed
SE-CȘ-O2	Serum	CȘ	O2	October 7, 2025	Discolored<br>Slightly hemolyzed
SE-CȘ-O3	Serum	CȘ	O3	October 7, 2025	Clear<br>Slightly yellow
SE-TNN-O1	Serum	TNN	O1	October 7, 2025	Clear<br>Slightly yellow
SE-UPCFBM-O1	Serum	UPCFBM	O1	October 7, 2025	Clear<br>Slightly yellow
Zone III includes the control area					
SE-T-O1	Serum	Tîrlișua	O1	October 10, 2025	Clear<br>Slightly yellow
SE-T-O2	Serum	Tîrlișua	O2	October 10–11, 2025	Clear<br>Slightly yellow
SE-T-O3	Serum	Tîrlișua	O3	October 10–11, 2025	Clear<br>Slightly yellow
SE-T-O4	Serum	Tîrlișua	O4	October 10–11, 2025	Clear<br>Slightly yellow
SE-T-O5	Serum	Tîrlișua	O5	October 10–11, 2025	Clear<br>Slightly yellow
SE-T-O6	Serum	Tîrlișua	O6	October 10–11, 2025	Clear<br>Slightly yellow
SE-T-O7	Serum	Tîrlișua	O7	October 10–11, 2025	Clear<br>Slightly yellow
SE-T-O8	Serum	Tîrlișua	O8	October 10–11, 2025	Clear<br>Slightly yellow
SE-T-O9	Serum	Tîrlișua	O9	October 10–11, 2025	Clear<br>Slightly yellow
SE-T-O10	Serum	Tîrlișua	O10	October 10–11, 2025	Clear<br>Slightly yellow

SE = Serum; TMBTP = Tăuții-Măgherăuș / Bozânta Mare tailings pods; RS = Recea / Săsar; TMN = Tăuții-Măgherăuș / Nistru; TMH = Tăuții-Măgherăuș / Herja; CI = Cicârlău / Ilba; CȘ = Cavnic / Șuior; Tăuții-Măgherăuș / Nistru = TNN; UPCFBM = UP Central Flotation / Bozânta Mare; Tîrlișua = T. O1 = Owner number 1; O2 = Owner number 2; O3 = Owner number 3; O4 = Owner number 4; O5 = Owner number 5; O6 = Owner number 6; O7 = Owner number 7; O8 = Owner number 8; O9 = Owner number 9; O10 = Owner number 10. Zone I n = 9 (serum samples); Zone II n = 27 (serum samples); Zone III n = 30 (serum samples). Sample code format: SE–[Site Code]–[Owner ID].

Table S4. Detailed Inventory of Equine Synovial Fluid Samples, Including Site, Owner, and Collection Date

Sample Code	Sample Type	Zone/The main sources of pollution	Owner	Collection Date	Observations
Zone I encompasses the areas of the former tailings ponds at Bozânta Mare, Săsar, and Nistru					
SF-TMBTP-O1	Synovial Fluid	TMBTP	O1	October 12, 2025	Normal clarity
SF-RS-O1	Synovial Fluid	RS	O1	October 12, 2025	Transparent, yellowish
SF-TMN-O1	Synovial Fluid	TMN	O1	October 12, 2025	Normal clarity
Zone II includes the areas of the former mines: Herja, Ilba, Șuior, Nistru, and UP Central Flotation					
SF-TMH-O1	Synovial Fluid	TMH	O1	October 12, 2025	Normal clarity
SF-TMH-O2	Synovial Fluid	TMH	O2	October 12, 2025	Transparent, yellowish
SF-CI-O1	Synovial Fluid	CI	O1	October 7, 2025	Clear fluid
SF-CI-O2	Synovial Fluid	CI	O2	October 7, 2025	Clear fluid
SF-CȘ-O1	Synovial Fluid	CȘ	O1	October 7, 2025	Transparent, yellowish
SF-CȘ-O2	Synovial Fluid	CȘ	O2	October 7, 2025	Transparent, yellowish
SF-CȘ-O3	Synovial Fluid	CȘ	O3	October 7, 2025	Clear fluid
SF-TNN-O1	Synovial Fluid	TNN	O1	October 7, 2025	Transparent, yellowish
SF-UPCFBM-O1	Synovial Fluid	UPCFBM	O1	October 7, 2025	Clear fluid
Zone III includes the control area					
SF-T-O1	Synovial Fluid	Tîrlișua	O1	October 7, 2025	Normal clarity
SF-T-O2	Synovial Fluid	Tîrlișua	O2	October 10-11, 2025	Clear, pale
SF-T-O3	Synovial Fluid	Tîrlișua	O3	October 10-11, 2025	Clear, pale
SF-T-O4	Synovial Fluid	Tîrlișua	O4	October 10-11, 2025	Clear fluid
SF-T-O5	Synovial Fluid	Tîrlișua	O5	October 10-11, 2025	Normal clarity
SF-T-O6	Synovial Fluid	Tîrlișua	O6	October 10-11, 2025	Clear, pale
SF-T-O7	Synovial Fluid	Tîrlișua	O7	October 10-11, 2025	Normal clarity
SF-T-O8	Synovial Fluid	Tîrlișua	O8	October 10-11, 2025	Clear fluid
SF-T-O9	Synovial Fluid	Tîrlișua	O9	October 10-11, 2025	Clear, pale
SF-T-O10	Synovial Fluid	Tîrlișua	O10	October 10-11, 2025	Normal clarity

SF = Synovial Fluid; TMBTP = Tăuții-Măgherăuș / Bozânta Mare tailings pods; RS = Recea / Săsar; TMN = Tăuții-Măgherăuș / Nistru; TMH = Tăuții-Măgherăuș / Herja; CI = Cicârlău / Ilba; CȘ = Cavnic / Șuior; Tăuții-Măgherăuș / Nistru = TNN; UPCFBM = UP Central

Flotation / Bozânta Mare; Tîrlișua = T. O1 = Owner number 1; O2 = Owner number 2; O3 = Owner number 3; O4 = Owner number 4; O5 = Owner number 5; O6 = Owner number 6; O7 = Owner number 7; O8 = Owner number 8; O9 = Owner number 9; O10 = Owner number 10. Zone I n = 9 (synovial fluid); Zone II n = 27 (synovial fluid); Zone III n = 30 (synovial fluid). Sample code format: SF–[Site Code]–[Owner ID].

Table S5. Detailed Inventory of Water Samples, Including Site, Owner, and Collection Date

Sample Code	Sample Type	Zone/The main sources of pollution	Owner	Collection Date	Observations
Zone I encompasses the areas of the former tailings ponds at Bozânta Mare, Săsar, and Nistru					
W-TMBTP-O1	Water	TMBTP	O1	March 12, 2025	Clear, slight odor
W-RS-O1	Water	RS	O1	March 12, 2025	Turbid, yellowish color
W-TMN-O1	Water	TMN	O1	March 12, 2025	Slightly cloudy
Zone II includes the areas of the former mines: Herja, Ilba, Șuior, Nistru, and UP Central Flotation					
W-TMH-O1	Water	TMH	O1	March 12, 2025	Clear
W-TMH-O2	Water	TMH	O2	March 12, 2025	Brownish tint
W-CI-O1	Water	CI	O1	March 7, 2025	Clear, no odor
W-CI-O2	Water	CI	O2	March 7, 2025	Slight sediment visibile
W-CȘ-O1	Water	CȘ	O1	March 7, 2025	Turbid, metallic smell
W-CȘ-O2	Water	CȘ	O2	March 7, 2025	Turbid, metallic smell
E-CȘ-O3	Water	CȘ	O3	March 7, 2025	Clear
W-TNN-O1	Water	TNN	O1	March 7, 2025	Slight oil sheen
W-UPCFBM-O1	Water	UPCFBM	O1	March 7, 2025	Clear
Zone III includes the control area					
W-T-O1	Water	Tîrlișua	O1	March 7, 2025	Clear, normal
W-T-O2	Water	Tîrlișua	O2	February 10-11, 2025	Clear
W-T-O3	Water	Tîrlișua	O3	February 10-11, 2025	Clear, no sediment
W-T-O4	Water	Tîrlișua	O4	February 10-11, 2025	Clear, odorless
W-T-O5	Water	Tîrlișua	O5	February 10-11, 2025	Clear
W-T-O6	Water	Tîrlișua	O6	February 10-11, 2025	Clear, fresh
W-T-O7	Water	Tîrlișua	O7	February 10-11, 2025	Clear, normal clarity
W-T-O8	Water	Tîrlișua	O8	February 10-11, 2025	Clear, slight turbidity
W-T-O9	Water	Tîrlișua	O9	February 10-11, 2025	Clear, no visible change
W-T-O10	Water	Tîrlișua	O10	February 10-11, 2025	Clear, normal appearance

W = Water; TMBTP = Tăuții-Măgherăuș / Bozânta Mare tailings pods; RS = Recea / Săsar; TMN = Tăuții-Măgherăuș / Nistru; TMH = Tăuții-Măgherăuș / Herja; CI = Cicârlău / Ilba; CȘ = Cavnic / Șuior; Tăuții-Măgherăuș / Nistru = TNN; UPCFBM = UP Central Flotation

/ Bozânta Mare; Tîrlișua = T. O1 = Owner number 1; O2 = Owner number 2; O3 = Owner number 3; O4 = Owner number 4; O5 = Owner number 5; O6 = Owner number 6; O7 = Owner number 7; O8 = Owner number 8; O9 = Owner number 9; O10 = Owner number 10. Zone I n = 9 (water samples); Zone II n = 27 (water samples); Zone III n = 30 (serum samples). Sample code format: W–[Site Code]–[Owner ID].

Table S6. Detailed Inventory of Vegetation Samples (Grass and Hay), Including Site, Owner, and Collection Date

Sample Code	Sample Type	Zone/The main sources of pollution	Owner	Collection Date	Observations
Zone I encompasses the areas of the former tailings ponds at Bozânta Mare, Săsar, and Nistru					
G-TMBTP-O1	Grass	TMBTP	O1	April 25, 2025	Green, healthy growth
H-TMBTP-O1	Hay			June 21, 2025	Dry, yellow edges
G-RS-O1	Grass	RS	O1	April 26, 2025	Active green vegetation typically observed in late spring
H-RS-O1	Hay			June 20, 2025	Harvested and dried plant biomass, typical for early summer
G-TMN-O1	Grass	TMN	O1	April 25, 2025	Green, healthy growth
H-TMN-O1	Hay			June 21, 2025	Dry, yellow edges
Zone II includes the areas of the former mines: Herja, Ilba, Șuior, Nistru, and UP Central Flotation					
G-TMH-O1	Grass	TMH	O1	April 23, 2025	Green, healthy growth
H-TMH-O1	Hay			June 21, 2025	Dry, yellow edges
G-TMH-O2	Grass	TMH	O2	April 26, 2025	Green, healthy growth
H-TMH-O2	Hay			June 20, 2025	Dry, yellow edges
G-CI-O1	Grass	CI	O1	April 23, 2025	Green, healthy growth
H-CI-O1	Hay			June 21, 2025	Dry, yellow edges
G-CI-O2	Grass	CI	O2	April 23, 2025	Strong, upright stems
H-CI-O2	Hay			June 21, 2025	Wilted, drooping leaves
G-CȘ-O1	Grass	CȘ	O1	April 15, 2025	Lush, green blades with high moisture
H-CȘ-O1	Hay			June 06, 2025	Dry, yellowing blades with stunted growth
G-CȘ-O2	Grass	CȘ	O2	April 15, 2025	Lush, green blades with high moisture

H-CȘ-O2	Hay			June 06, 2025	Dry, yellowing blades with stunted growth
G-CȘ-O3	Grass	CȘ	O3	April 15, 2025	Soft, pliable leaves that recover after trampling
H-CȘ-O3	Hay			June 06, 2025	Brittle, curling leaves that snap easily
G-TNN-O1	Grass	TNN	O1	April 15, 2025	Green, healthy growth
H-TNN-O1	Hay			June 06, 2025	Dry, yellow edges
G-UPCFBM-O1	Grass	UPCFBM	O1	April 15, 2025	Green, healthy growth
H-UPCFBM-O1	Hay			June 06, 2025	Dry, yellow edges
Zone III includes the control area					
G-T-O1	Grass	Tîrlișua	O1	April 17, 2025	Strong, upright stems
H-T-O1	Hay			June 11, 2025	Wilted, drooping leaves
G-T-O2	Grass	Tîrlișua	O2	April 17, 2025	Strong, upright stems
H-T-O2	Hay			June 11, 2025	Wilted, drooping leaves
G-T-O3	Grass	Tîrlișua	O3	April 17, 2025	Strong, upright stems
H-T-O3	Hay			June 11, 2025	Wilted, drooping leaves
G-T-O4	Grass	Tîrlișua	O4	April 17, 2025	Strong, upright stems
H-T-O4	Hay			June 11, 2025	Wilted, drooping leaves
G-T-O5	Grass	Tîrlișua	O5	April 17, 2025	Strong, upright stems
H-T-O5	Hay			June 11, 2025	Wilted, drooping leaves
G-T-O6	Grass	Tîrlișua	O6	April 17, 2025	Strong, upright stems
H-T-O6	Hay			June 11, 2025	Wilted, drooping leaves
G-T-O7	Grass	Tîrlișua	O7	April 17, 2025	Strong, upright stems
H-T-O7	Hay			June 11, 2025	Wilted, drooping leaves

G-T-O8	Grass	Tîrlișua	O8	April 17, 2025	Strong, upright stems
H-T-O8	Hay			June 11, 2025	Wilted, drooping leaves
G-T-O9	Grass	Tîrlișua	O9	April 17, 2025	Strong, upright stems
H-T-O9	Hay			June 11, 2025	Wilted, drooping leaves
G-T-O10	Grass	Tîrlișua	O10	April 17, 2025	Strong, upright stems
H-T-O10	Hay			June 11, 2025	Wilted, drooping leaves

G = Grass; H = Hay; TMBTP = Tăuții-Măgherăuș / Bozânta Mare tailings pods; RS = Recea / Săsar; TMN = Tăuții-Măgherăuș / Nistru; TMH = Tăuții-Măgherăuș / Herja; CI = Cicârlău / Ilba; CȘ = Cavnic / Șuior; Tăuții-Măgherăuș / Nistru = TNN; UPCFBM = UP Central Flotation / Bozânta Mare; Tîrlișua = T. O1 = Owner number 1; O2 = Owner number 2; O3 = Owner number 3; O4 = Owner number 4; O5 = Owner number 5; O6 = Owner number 6; O7 = Owner number 7; O8 = Owner number 8; O9 = Owner number 9; O10 = Owner number 10. Zone I n = 18 (grass (9) and hay (9) samples); Zone II n = 54 (grass (27) and hay (27) samples); Zone III n = 60 (grass (30) and hay (30) samples). Sample code format: G/H–[Site Code]–[Owner ID].

Table S7. Detailed Inventory of Equine Feed Samples (Concentrates), Including Site, Owner, and Collection Date

Sample Code	Sample Type	Zone/The main sources of pollution	Owner	Collection Date	Observations
Zone III includes the control area					
C-T-O1	Concentrates	Tîrlișua	O3	February 10-11, 2025	Uniform grind, no visible impurities
C-T-O2	Concentrates	Tîrlișua	O5	February 10-11, 2025	Dry, golden-yellow, consistent texture
C-T-O4	Concentrates	Tîrlișua	O6	February 10-11, 2025	Slightly coarse, natural appearance
C-T-O8	Concentrates	Tîrlișua	O7	February 10-11, 2025	Fine texture, no off-colors

In total, twelve concentrate samples were collected from the control area of Tîrlișua, corresponding to owners 1, 2, 4, and 8. Although referred to as “concentrates,” the samples consisted of locally grown corn (*Zea mays*) intended as standardized equine feed. The corn was administered to horses during field work periods, for approximately 4 to 8 weeks, at a frequency of five days per week, in order to simulate typical rural feeding practices under uncontaminated conditions. Sample code format: C–[Site Code]–[Owner ID].

Table S8. Detailed Inventory of Soil Samples, Including Site, Owner, and Collection Date

Sample Code	Sample Type	Zone/The main sources of pollution	Owner	Collection Date	Observations
Zone I encompasses the areas of the former tailings ponds at Bozânta Mare, Săsar, and Nistru					
S-TMBTP-O1	Soil	TMBTP	O1	March 12, 2025	Slightly yellow soil
S-RS-O1	Soil	RS	O1	March 12, 2025	Slightly yellow soil
S-TMN-O1	Soil	TMN	O1	March 12, 2025	Slightly yellow soil
Zone II includes the areas of the former mines: Herja, Ilba, Șuior, Nistru, and UP Central Flotation					
S-TMH-O1	Soil	TMH	O1	March 12, 2025	Slightly yellow soil, friable
S-TMH-O2	Soil	TMH	O2	March 12, 2025	Slightly yellow soil, moist
S-CI-O1	Soil	CI	O1	March 7, 2025	Dark grey soil, slightly compact
S-CI-O2	Soil	CI	O2	March 7, 2025	Slightly yellow soil, friable
S-CȘ-O1	Soil	CȘ	O1	March 7, 2025	Dark grey soil, moist
S-CȘ-O2	Soil	CȘ	O2	March 7, 2025	Greyish soil, compact
S-CȘ-O3	Soil	CȘ	O3	March 7, 2025	Slightly yellow soil, friable
S-TNN-O1	Soil	TNN	O1	March 7, 2025	Slightly yellow soil, sandy texture
S-UPCFBM-O1	Soil	UPCFBM	O1	March 7, 2025	Slightly yellow soil, dry surface
Zone III includes the control area					
S-T-O1	Soil	Tîrlișua	O1	March 7, 2025	Slightly yellow soil, friable
S-T-O2	Soil	Tîrlișua	O2	February 10-11, 2025	Slightly yellow soil, moist
S-T-O3	Soil	Tîrlișua	O3	February 10-11, 2025	Yellowish-brown soil, friable
S-T-O4	Soil	Tîrlișua	O4	February 10-11, 2025	Slightly yellow soil, sandy texture
S-T-O5	Soil	Tîrlișua	O5	February 10-11, 2025	Slightly yellow soil, dry surface
S-T-O6	Soil	Tîrlișua	O6	February 10-11, 2025	Light brown soil, compact
S-T-O7	Soil	Tîrlișua	O7	February 10-11, 2025	Slightly yellow soil, friable
S-T-O8	Soil	Tîrlișua	O8	February 10-11, 2025	Slightly yellow soil, moist

SE-T-O9	Soil	Tîrlișua	O9	February 10-11, 2025	Light brown soil, sandy texture
SE-T-O10	Soil	Tîrlișua	O10	February 10-11, 2025	Slightly yellow soil, friable

S = Soil; TMBTP = Tăuții-Măgherăuș / Bozânta Mare tailings ponds; RS = Recea / Săsar; TMN = Tăuții-Măgherăuș / Nistru; TMH = Tăuții-Măgherăuș / Herja; CI = Cicârlău / Ilba; CȘ = Cavnic / Șuior; TNN = Tăuții-Măgherăuș / Nistru (tailings ponds area); UPCFBM = UP Central Flotation / Bozânta Mare; T = Tîrlișua (control area). O1 = Owner number 1; O2 = Owner number 2; O3 = Owner number 3; O4 = Owner number 4; O5 = Owner number 5; O6 = Owner number 6; O7 = Owner number 7; O8 = Owner number 8; O9 = Owner number 9; O10 = Owner number 10. Zone I n = 9 (soil samples); Zone II n = 27 (soil samples); Zone III n = 30 (soil samples). Sample code format: S–[Site Code]–[Owner ID].

Table S9. The operational program of the Milestone START D Microwave Digestion System, which regulates the parameters for sample disaggregation and digestion

Step	Target Temp (°C)	Pressure Max. (psi)	Temperature Ramp (min.)	Hold Time (min.)	Power (%)
Soil[1]					
1.	200	800	10	5	100
2.	220	800	15	20	100
3.	35-40	800	-	55 min. cooling	-
Dried Hay/Green grass[2]					
1.	85	800	4	5	100
2.	145	800	9	3	100
3.	200	800	4	3	100
4.	200	800	14	10	100
5.	35-40	800	-	55 min. cooling	-
Water[3]					
1.	200	800	10	5	100
2.	200	800	20	15	100
3.	35-40	800	-	55 min. cooling	-
Concentrate[4]					
1.	200	800	10	5	100
2.	200	800	20	15	100
3.	35-40	800	-	55 min. cooling	-
Hair[5]					
1.	85	800	2	3	100
2.	135	800	4	3	100
3.	200	800	5	5	100
4.	200	800	15	20	100
5.	35-40	800	-	55 min. cooling	-
Hoof/Synovial fluid[6]					
1.	85	800	4	5	100
2.	145	800	9	3	100
3.	200	800	4	3	100
4.	220	800	14	10	100
5.	35-40	800	-	55 min. cooling	-
Blood serum[7]					
1.	85	800	2	3	100
2.	135	800	4	3	100
3.	230	800	5	5	100

4.	230	800	15	20	100
5.	35-40	800	-	55 min. cooling	-

List of standards used for sample disaggregation according to the Milestone START D Microwave Digestion System program. DG_EN-12 Soil[1]; DG_EN-05 Grass[2]; DG_EN-18 Wastewater[3]; DG-FO-61 Cereals/DG_AG-04 Maize[4]; DG-CL-10 Hair[5]; DG-CL-02 Animal tissue[6]; DG-CL-03 Blood[7].

Table S10. The instrumental settings (a) and data acquisition parameters (b) of the ICP-MS system, which define the operating conditions and analytical procedures for precise metal quantification

**(a) Instrumental parameters**		**(b) Data acquisition parameters for quantitative mode**	
RF power/W	1.4 kW	Measuring mode	Standard (Ar 5.0)<br>Q Cell (Collision Cell) (He 6.0)
Argon (Ar) gas flow, Helium (He) gas flow		Point per peak	3
Nebulizer	1.0 L/min.	Scans/Replicate	7
Plasma gas low rate (Ar 5.0)	18.0 L/min.	Replicate/Sample	7
Auxiliary gas flow rate (He 6.0)	0.20 L/min.		
Lens voltage	37 V	Dwell time (ms)	3
Mirror lens right	32 V		
Mirror lens bottom	31 V		
Sample uptake rate	90 s	Integration time	1-5 ms
Temperature spray chamber		2.10 °C	
Background correction		2 points/peak	
Injector tube		quartz 2-mm id	
Sample cone		Sample Cone 4450	
Skimmer cone		Ni – Skimmer iCAP Q 0.5 mm insert version	
Nebulizer		MicroMist Nebulizer 0.4 mL/min.	

Table S11. Instrumental conditions for the determination of each element using ICP-MS technique.

**Element**	**Correlation coefficient**	**LoD (μg/L)**	**LoQ (μg/L)**	**BEC (μg/L)**
^{64}Cu	0.9997	0.035	0.139	0.236
^{65}Zn	0.9999	0.079	1.203	1.310
^{208}Pb	0.9996	0.151	0.231	0.649
^{111}Cd	0.9997	0.007	0.069	0.0031
^{60}Ni	0.9997	0.045	0.181	0.096
^{59}Co	0.9997	0.051	0.136	0.152
^{75}As	0.9999	0.006	0.743	0.018
^{52}Cr	0.9999	1.607	5.533	0.637
^{201}Hg	0.9999	0.043	0.137	0.128

LoD = Detection limit; LoQ = Quantification limit; BEC = Background equivalent concentration.

Table S12. Validation parameters of the analytical procedure for the determination of heavy metals (mane and tail hair samples)

**Element**	**The result declared by the manufacturer**	**The results obtained in our research**	**Recovery (%)**	**Uncertainty (%)**
^{64}Cu (mg/kg)[b]	11.5 ± 0.6	11.2 ± 0.7	97.4	15
^{65}Zn (mg/kg)[b]	160 ± 5	165 ± 6	103.1	14

^{208}Pb (mg/kg)[a]	5.45 ± 0.20	5.22 ± 0.18	95.8	13
^{111}Cd (mg/kg)[a]	0.190 ± 0.011	0.183 ± 0.010	96.3	12
^{60}Ni (mg/kg)[b]	1.72 ± 0.08	1.64 ± 0.07	95.3	17
^{59}Co (mg/kg)[b]	0.410 ± 0.025	0.427 ± 0.030	104.1	18
^{75}As (mg/kg)[a]	0.145 ± 0.010	0.150 ± 0.011	103.4	19
^{52}Cr (mg/kg)[b]	2.60 ± 0.12	2.48 ± 0.10	95.4	16
^{201}Hg (mg/kg)[a]	0.325 ± 0.022	0.310 ± 0.021	95.4	11

Notes: a – Data obtained using IAEA-085 (Human Hair) certified values. b – Elements determined by ICP–MS (Thermo Scientific iCAP Q) in collision cell mode with He gas. All recoveries are within the acceptable range of 90–110%, confirming the accuracy and reliability of the analytical procedure for hair matrices.

Table S13. Validation parameters of the analytical procedure for the determination of heavy metals (hoof samples)

**Element**	**The result declared by the manufacturer**	**The results obtained in our research**	**Recovery (%)**	**Uncertainty (%)**
^{64}Cu (mg/kg)[b]	4.32 ± 0.18	4.28 ± 0.22	98.4	15
^{65}Zn (mg/kg)[b]	98.6 ± 2.9	101.2 ± 3.6	102.6	14
^{208}Pb (mg/kg)[a]	0.48 ± 0.02	0.465 ± 0.019	96.5	12
^{111}Cd (mg/kg)[a]	0.083 ± 0.005	0.079 ± 0.004	94.0	13
^{60}Ni (mg/kg)[b]	0.93 ± 0.04	0.888 ± 0.038	96.0	17
^{59}Co (mg/kg)[b]	0.21 ± 0.02	0.215 ± 0.016	103.4	18
^{75}As (mg/kg)[a]	0.136 ± 0.009	0.141 ± 0.010	105.2	19
^{52}Cr (mg/kg)[b]	1.83 ± 0.07	1.78 ± 0.08	96.2	16
^{201}Hg (mg/kg)[a]	0.028 ± 0.002	0.027 ± 0.002	96.4	11

Notes: a – Data obtained using BCR-185R (Bovine Liver) certified values. b – Elements determined by ICP–MS (Thermo Scientific iCAP Q) in collision cell mode with He gas. All recoveries are within the acceptable range of 90–110%, confirming the accuracy and reliability of the analytical procedure for keratinized tissue matrices (hoof).

Table S14. Validation parameters of the analytical procedure for the determination of heavy metals (Caprine Blood and Synovial Fluid)

**Element**	**Certified reference material analysis**		**Validation parameters**	
	The result declared by de manufacture	The results obtained in our research	Recovery (%)	Uncertainty (%)
^{64}Cu (μg/L)[b]	1582 ± 96	1586 ± 102	102.78	21
^{65}Zn (μg/L)[b]	880 ± 24	856 ± 11	102.08	19
^{208}Pb (μg/dL)[a]	0.415 ± 0.0012	0.478 ± 0.0001	96.0	11
^{111}Cd (μg/L)[a]	0.0319 ± 0.0062	0.0317 ± 0.005	98.3	13
^{60}Ni (μg/L)[b]	0.99 ± 0.24	0.98 ± 0.05	91.5	21
^{59}Co (μg/L)[b]	1.22 ± 0.04	1.18 ± 0.06	99.6	22
^{75}As (μg/L)[a]	21.67 ± 0.15	23.04 ± 0.21	95.7	19
^{52}Cr (μg/L)[b]	0.33 ± 0.06	0.36 ± 0.03	101.2	22
^{201}Hg (μg/kg)[a]	0.017 ± 0.011	0.016 ± 0.01	98.2	13

- [a] SRM – 955c Standard Reference Material Toxic Metals in Caprine Blood; [b] SRM – 1598a Standard Reference Material Inorganic Constituents in Animal Serum.

Table S15. Validation parameters of the analytical procedure for the determination of heavy metals (water samples)

Element	The result declared by the manufacturer	The results obtained in our research	Recovery (%)	Uncertainty (%)
^{64}Cu (μg/L)[b]	22.3 ± 1.1	21.8 ± 1.5	97.8	14
^{65}Zn (μg/L)[b]	72.9 ± 1.7	74.2 ± 2.0	101.8	15
^{208}Pb (μg/L)[a]	19.6 ± 0.8	18.9 ± 0.7	96.4	12
^{111}Cd (μg/L)[a]	0.612 ± 0.022	0.595 ± 0.025	97.2	13
^{60}Ni (μg/L)[b]	59.2 ± 2.3	57.5 ± 1.9	97.1	18
^{59}Co (μg/L)[b]	2.38 ± 0.09	2.44 ± 0.12	102.5	17
^{75}As (μg/L)[a]	26.7 ± 1.1	27.4 ± 1.3	102.6	19
^{52}Cr (μg/L)[b]	18.5 ± 0.7	17.9 ± 0.6	96.8	16
^{201}Hg (μg/L)[a]	0.010 ± 0.001	0.0095 ± 0.0008	95.0	11

Notes: a – Data obtained using NIST SRM 1643f (Trace Elements in Water) certified values. b – Elements determined by ICP–MS (Thermo Scientific iCAP Q) in collision cell mode with He gas. All recoveries are within the acceptable range of 90–110%, confirming the accuracy and reliability of the analytical procedure.

Table S16. Validation parameters of the analytical procedure for determination of heavy metals (green grass)

**Element**	**Certified reference material analysis**		**Validation parameters**	
	The result declared by de manufacture	The results obtained in own research	Recovery (%)	Uncertainty (%)
^{64}Cu (mg/kg)[a]	4.70 ± 0.14	4.42 ± 0.31	96.87	23
^{65}Zn (mg/kg)[a]	30.94 ± 0.55	31.84 ± 0.86	100.12	17
^{208}Pb (mg/kg)[b]	0.167 ± 0.015	0.164 ± 0.003	96.78	22
^{111}Cd (mg/kg)[a]	1.517 ± 0.027	1.687 ± 0.147	90.32	23
^{60}Ni (mg/kg)[a]	1.582 ± 0.041	1.784 ± 0.084	98.70	18
^{59}Co (mg/kg)[a]	0.5773 ± 0.071	0.6541 ± 0.321	107.18	24
^{75}As (mg/kg)[a]	0.1126 ± 0.024	0.1126 ± 0.062	117.89	16
^{52}Cr (mg/kg)[a]	1.988 ± 0.034	2.124 ± 0.070	92.69	21
^{201}Hg (mg/kg)[a]	0.0341 ± 0.0015	0.023 ± 0.017	95.84	10

[a] NIST – 1573a Tomato Leaves Standard Reference Materials; [b] NIST – 1575a Pine Needles (*Pinus taeda*) Standard Reference Materials

Table S17. Validation parameters of the analytical procedure for the determination of heavy metals (concentrate feed – maize-based samples)

Element	The result declared by the manufacturer	The results obtained in our research	Recovery (%)	Uncertainty (%)
^{64}Cu (mg/kg)[b]	3.68 ± 0.14	3.61 ± 0.19	98.1	16
^{65}Zn (mg/kg)[b]	24.0 ± 0.8	25.2 ± 1.3	105.0	15
^{208}Pb (mg/kg)[a]	0.162 ± 0.012	0.155 ± 0.008	95.7	13

^{111}Cd (mg/kg)[a]	0.069 ± 0.004	0.067 ± 0.004	97.1	14
^{60}Ni (mg/kg)[b]	1.56 ± 0.07	1.49 ± 0.06	95.5	17
^{59}Co (mg/kg)[b]	0.47 ± 0.03	0.49 ± 0.02	104.3	18
^{75}As (mg/kg)[a]	0.102 ± 0.009	0.108 ± 0.010	105.9	19
^{52}Cr (mg/kg)[b]	1.92 ± 0.08	1.83 ± 0.07	95.3	16
^{201}Hg (mg/kg)[a]	0.032 ± 0.003	0.030 ± 0.002	95.2	11

Notes: a – Data obtained using ERM®-BC382 Maize Flour (JRC, European Commission, Belgium) certified values. b – Elements determined by ICP–MS (Thermo Scientific iCAP Q) in collision cell mode with He gas. All recoveries are within the acceptable range of 90–110%, confirming the accuracy and reliability of the analytical procedure for maize-based concentrate feed.

Table S18. Validation parameters of the analytical procedure for the determination of heavy metals (soil)

**Element**	**Certified reference material analysis**		**Validation parameters**	
	The result declared by de manufacture	The results obtained in our research	Recovery (%)	Uncertainty (%)
^{64}Cu (mg/kg)	34.6 ± 0.7	32.45 ± 2.78	96.7	19
^{65}Zn (mg/kg)	106 ± 3	100.1 ± 3.04	95.2	18
^{208}Pb (mg/kg)	18.9 ± 0.5	19.16 ± 0.84	96.0	11
^{111}Cd (mg/kg)	0.38 ± 0.01	0.37 ± 0.01	99.1	12
^{60}Ni (mg/kg)	88 ± 5	85.8 ± 2.36	85.3	16
^{59}Co (mg/kg)	13.4 ± 0.7	13.97 ± 0.75	95.5	21
^{75}As (mg/kg)	17.7 ± 0.8	16.7 ± 0.94	92.6	22
^{52}Cr (mg/kg)	103 ± 4	101.21 ± 2.36	98.8	18
^{201}Hg (mg/kg)	1.4 ± 0.08	1.29 ± 0.02	98.7	13

- SRM – 2709a Standard Reference Material „San Joaquin Soil" Baseline Trace Element Concentration.

Table S19. Limits of Heavy Metals in Water – For Human Consumption, Surface Water, and Animal Use (Including Horses)

**Metal**	**Drinking Water (mg/L) (HG 102/2022, Dir. 2020/2184)** [51,52]	**Surface Water Class I (mg/L) (Ord. 161/2006, Dir. 2000/60/CE)** [53–55]	**Water for Animals (mg/L) (FAO, CCME, NRC)** [56–58]	**Observations**
Cu	2.0	0.01	1.0 – 5.0	Essential trace element; toxic >1 mg/L in young animals.
Zn	Not specified (gust >3–5 mg/L)	0.03	5.0 – 25.0	Affects taste; toxic >100 mg/L.
Pb	0.005	0.01	0.1	Highly toxic, accumulates in bones and soft tissues.
Cd	0.003	0.001	0.01 – 0.05	Carcinogenic, kidney damage at low levels.
Ni	0.020	0.01	1.0	Irritation and organ toxicity at high doses.
Co	Not specified	0.01	1.0	Needed for B12 synthesis; toxic >1 mg/L.

As	0.010	0.01	0.05	Chronic exposure can lead to cancer and skin lesions.
Cr	0.050	0.05	0.05 – 0.1	Cr VI more toxic than Cr III; liver and kidney damage.
Hg	0.001	0.0002	0.001	Extremely toxic; neurological and kidney effects.

toxics MDPI

Table S20. Assessment of Heavy metals Contamination Across Three Zone Based on Legal Drinking Water Limits: A Descriptive Comparison

**Zone I**	**Cu Min (mg/L)**	**Cu Mean (mg/L)**	**Cu max (mg/L)**	**Zn Min (mg/L)**	**Zn Mean (mg/L)**	**Zn max (mg/L)**	**Pb Min (mg/L)**	**Pb Mean (mg/L)**	**Pb max (mg/L)**
Legal limits		2.0 mg Cu/L			> 3-5 mg Zn/L			0.005 mg Pb/L	
	1.92	3.50	5.54	1.98	2.81	3.33	0.03	0.04	0.05
	Cd Min (mg/L)	Cd Mean (mg/L)	Cd max (mg/L)	Ni Min (mg/L)	Ni Mean (mg/L)	Ni max (mg/L)	Co Min (mg/L)	Co Mean (mg/L)	Co max (mg/L)
Legal limits		0.003 mg Cd/L			0.020 mg Ni/L			Not specified	
	BLD	BLD	BLD	0.09	0.10	0.11	0.07	0.16	0.23
	As Min (mg/L)	As Mean (mg/L)	As max (mg/L)	Cr Min (mg/L)	Cr Mean (mg/L)	Cr max (mg/L)	Hg Min (mg/L)	Hg Mean (mg/L)	Hg max (mg/L)
Legal limits		0.010 mg As/L			0.050 mg Cr/L			0.001 Hg/L	
	BLD	BLD	BLD	0.01	0.02	0.05	BLD	BLD	BLD
Zone II	Cu Min (mg/L)	Cu Mean (mg/L)	Cu max (mg/L)	Zn Min (mg/L)	Zn Mean (mg/L)	Zn max (mg/L)	Pb Min (mg/L)	Pb Mean (mg/L)	Pb max (mg/L)
Legal limits		2.0 mg Cu/L			> 3-5 mg Zn/L			0.005 mg Pb/L	
	0.91	3.10	7.93	2.52	3.77	5.23	0.02	0.04	0.09
	Cd Min (mg/L)	Cd Mean (mg/L)	Cd max (mg/L)	Ni Min (mg/L)	Ni Mean (mg/L)	Ni max (mg/L)	Co Min (mg/L)	Co Mean (mg/L)	Co max (mg/L)
Legal limits		0.003 mg Cd/L			0.020 mg Ni/L			Not specified	
	BLD	BLD	BLD	0.07	0.11	0.16	0.05	0.11	0.36
	As Min (mg/L)	As Mean (mg/L)	As max (mg/L)	Cr Min (mg/L)	Cr Mean (mg/L)	Cr max (mg/L)	Hg Min (mg/L)	Hg Mean (mg/L)	Hg max (mg/L)
Legal limits		0.010 mg As/L			0.050 mg Cr/L			0.001 Hg/L	
	BLD	BLD	BLD	0.01	0.02	0.04	BLD	BLD	BLD
Zone III	Cu Min (mg/L)	Cu Mean (mg/L)	Cu max (mg/L)	Zn Min (mg/L)	Zn Mean (mg/L)	Zn max (mg/L)	Pb Min (mg/L)	Pb Mean (mg/L)	Pb max (mg/L)
Legal limits		2.0 mg Cu/L			> 3-5 mg Zn/L			0.005 mg Pb/L	
	0.018	0.020	0.021	0.011	0.013	0.014	0.012	0.013	0.014
	Cd Min (mg/L)	Cd Mean (mg/L)	Cd max (mg/L)	Ni Min (mg/L)	Ni Mean (mg/L)	Ni max (mg/L)	Co Min (mg/L)	Co Mean (mg/L)	Co max (mg/L)
Legal limits		0.003 mg Cd/L			0.020 mg Ni/L			Not specified	
	BLD	BLD	BLD	0.018	0.019	0.020	0.002	0.003	0.003

	As Min (mg/L)	As Mean (mg/L)	As max (mg/L)	Cr Min (mg/L)	Cr Mean (mg/L)	Cr max (mg/L)	Hg Min (mg/L)	Hg Mean (mg/L)	Hg max (mg/L)
Legal limits		0.010 mg As/L			0.050 mg Cr/L			0.001 mg Hg/L	
	BLD	BLD	BLD	0.001	0.001	0.002	BLD	BLD	BLD

Min = minimum; Mean = arithmetic mean; Max = maximum; BLD = Below Detection Limit. All concentrations are expressed in milligrams per liter (mg/L). Legal thresholds correspond to the maximum permissible concentrations for drinking water established by HG 102/2022 (Romania) and Directive (EU) 2020/2184 of the European Parliament and of the Council on the quality of water intended for human consumption. Zn guideline values refer to acceptable taste thresholds rather than toxicological limits (>3–5 mg/L). Analytical precision (RSD) was generally <6% for Cu and Zn, with higher variability for Pb and Co near detection limits. These regulatory thresholds serve as reference benchmarks for assessing compliance and evaluating potential risks to environmental and public health.

Table S21. Legal Limits for Heavy Metals in Grass and Hay (Feed Use)

Heavy Metal	Maximum Limit (mg/kg)	Applicable To	Legal Source / Reference	Remarks
Lead (Pb)	10.0	Feed materials (e.g., grass, hay)	Directive 2002/32/EC, Annex I	Limit expressed on dry matter basis
Cadmium (Cd)	1.0	Feed materials (e.g., grass, hay)	Directive 2002/32/EC, Annex I	Includes forage and pasture plants
Mercury (Hg)	0.1	Feed materials (e.g., grass, hay)	Directive 2002/32/EC, Annex I	Rarely exceeds threshold in open fields
Arsenic (As)	2.0	Feed materials (e.g., grass, hay)	Directive 2002/32/EC, Annex I	May occur near mining zones or contaminated sites
Fluorine (F)	30.0	Complete/complementary feed for horses	Directive 2002/32/EC, Annex I	Specific to adult horses; excessive amounts affect bones and teeth

All limits are expressed in milligrams per kilogram (mg/kg) of dry matter. Values represent the maximum permissible concentrations for feed materials (grass, hay, and forage) according to Directive 2002/32/EC of the European Parliament and of the Council on undesirable substances in animal feed (Annex I, consolidated version). These thresholds are established to protect animal health and prevent metal accumulation in the food chain.

Table S22. Assessment of Heavy Metal Accumulation in Grass and Hay Samples Across Three Zones Based on Legal Feed and Food Safety Limits: A Descriptive Comparison

**Zone I**	**Cu Min (mg/kg)**	**Cu Mean (mg/ kg)**	**Cu max (mg/ kg)**	**Zn Min (mg/ kg)**	**Zn Mean (mg/ kg)**	**Zn max (mg/L)**	**Pb Min (mg/ kg)**	**Pb Mean (mg/ kg)**	**Pb max (mg/ kg)**
Legal limits		-			-			10 mg Pb/kg	
					Grass				
	11.43	12.82	14.91	119.26	134.49	147.54	1.25	1.62	2.12
					Hay				
	9.50	10.66	12.40	96.93	109.56	118.33	1.39	2.48	4.04
	Cd Min (mg/ kg)	Cd Mean (mg/kg)	Cd max (mg/kg)	Ni Min (mg/kg)	Ni Mean (mg/kg)	Ni max (mg/kg)	Co Min (mg/kg)	Co Mean (mg/kg)	Co max (mg/kg)
Legal limits		1.00 mg Cd/kg			-			-	
					Grass				
	0.27	0.36	0.45	BLD	0.52	0.15	BLD	BLD	BLD
					Hay				
	0.20	0.39	0.65	BLD	0.38	1.13	BLD	BLD	BLD
	As Min (mg/kg)	As Mean (mg/kg)	As max (mg/kg)	Cr Min (mg/kg)	Cr Mean (mg/kg)	Cr max (mg/kg)	Hg Min (mg/kg)	Hg Mean (mg/kg)	Hg max (mg/kg)
Legal limits		2.00 mg As/kg			-			0.10 mg/Hg/kg	
					Grass				
	BLD	BLD	BLD	BLD	BLD	BLD	BLD	BLD	BLD
					Hay				
	BLD	BLD	BLD	BLD	BLD	BLD	BLD	BLD	BLD
Zone II	Cu Min (mg/kg)	Cu Mean (mg/ kg)	Cu max (mg/ kg)	Zn Min (mg/ kg)	Zn Mean (mg/ kg)	Zn max (mg/L)	Pb Min (mg/ kg)	Pb Mean (mg/ kg)	Pb max (mg/ kg)
Legal limits		-			-			10 mg Pb/kg	
					Grass				
	1.69	6.35	12.51	113.22	140.60	167.46	3.48	5.61	7.91
					Hay				
	1.61	5.54	11.48	102.39	124.02	150.89	2.92	5.06	7.21

	Cd Min (mg/ kg)	Cd Mean (mg/kg)	Cd max (mg/kg)	Ni Min (mg/kg)	Ni Mean (mg/kg)	Ni max (mg/kg)	Co Min (mg/kg)	Co Mean (mg/kg)	Co max (mg/kg)
Legal limits		1.00 mg Cd/kg			-			-	
					Grass				
	0.20	0.34	0.47	-	0.79	1.84	BLD	BLD	BLD
					Hay				
	0.16	0.27	0.37	-	0.61	1.44	BLD	BLD	BLD
	As Min (mg/kg)	As Mean (mg/kg)	As max (mg/kg)	Cr Min (mg/kg)	Cr Mean (mg/kg)	Cr max (mg/kg)	Hg Min (mg/kg)	Hg Mean (mg/kg)	Hg max (mg/kg)
Legal limits		2.00 mg As/kg			-			0.10 mg/Hg/kg	
					Grass				
	BLD	BLD	BLD	BLD	BLD	BLD	BLD	BLD	BLD
					Hay				
	BLD	BLD	BLD	BLD	BLD	BLD	BLD	BLD	BLD
Zone III	Cu Min (mg/kg)	Cu Mean (mg/ kg)	Cu max (mg/ kg)	Zn Min (mg/ kg)	Zn Mean (mg/ kg)	Zn max (mg/L)	Pb Min (mg/ kg)	Pb Mean (mg/ kg)	Pb max (mg/ kg)
Legal limits		-			-			10 mg Pb/kg	
					Grass				
	0.16	0.37	0.54	12.56	21.94	29.11	0.16	0.54	0.89
					Hay				
	0.26	0.31	0.34	20.16	21.73	23.56	0.16	0.31	0.42
	Cd Min (mg/ kg)	Cd Mean (mg/kg)	Cd max (mg/kg)	Ni Min (mg/kg)	Ni Mean (mg/kg)	Ni max (mg/kg)	Co Min (mg/kg)	Co Mean (mg/kg)	Co max (mg/kg)
Legal limits		1.00 mg Cd/kg			-			-	
					Grass				
	0.05	0.06	0.07	-	-	-	BLD	BLD	BLD
					Hay				
	0.04	0.04	0.05	-	-	-	BLD	BLD	BLD
	As Min (mg/kg)	As Mean (mg/kg)	As max (mg/kg)	Cr Min (mg/kg)	Cr Mean (mg/kg)	Cr max (mg/kg)	Hg Min (mg/kg)	Hg Mean (mg/kg)	Hg max (mg/kg)
Legal limits		2.00 mg As/kg			-			0.10 mg/Hg/kg	
					Grass				
	BLD	BLD	BLD	BLD	BLD	BLD	BLD	BLD	BLD

Hay									
	BLD	BLD	BLD	BLD	BLD	BLD	BLD	BLD	BLD

Min = minimum; Mean = arithmetic mean; Max = maximum; BLD = Below Detection Limit. All concentrations are expressed on a dry-weight basis (mg/kg). Legal thresholds correspond to the maximum permissible concentrations for feed and food materials established by Directive 2002/32/EC (Annex I) on undesirable substances in animal feed and by Regulation (EC) No 1881/2006 on maximum levels for certain contaminants in foodstuffs. In the absence of specific legal thresholds for heavy metals in corn-based concentrates intended for equine feed, the evaluation was performed by reference to the maximum permissible limits for forage materials (grass and hay) listed in Directive 2002/32/EC, Annex I: Pb – 10.0 mg/kg, Cd – 1.0 mg/kg, Hg – 0.1 mg/kg, As – 2.0 mg/kg, and F – 30.0 mg/kg (for complete or complementary feeds for adult horses). Analytical precision (RSD) was generally <6% for Cu and Zn, with higher variability for Pb and Co near detection limits. These thresholds serve as comparative benchmarks for assessing compliance and evaluating potential risks to feed and food safety.

toxics MDPI

Table S23. Assessment of Heavy Metal Accumulation in Corn-Based Concentrate Samples from the Control Zone: Comparison with Legal Feed and Food Safety Limits

Zone III	Cu Min (mg/kg)	Cu Mean (mg/kg)	Cu max (mg/kg)	Zn Min (mg/kg)	Zn Mean (mg/kg)	Zn max (mg/kg)	Pb Min (mg/kg)	Pb Mean (mg/kg)	Pb max (mg/kg
Legal limits		-			-			10.0 mg Pb/kg	
	1.77	1.80	1.82	27.56	28.05	28.45	0.03	0.04	0.04
	Cd Min (mg/kg)	Cd Mean (mg/kg)	Cd max (mg/kg)	Ni Min (mg/kg)	Ni Mean (mg/kg)	Ni max (mg/kg)	Co Min (mg/kg)	Co Mean (mg/kg)	Co max (mg/kg)
Legal limits		1.0 mg Cd/kg			-			-	
	BLD	BLD	BLD	0.09	0.10	0.11	0.04	0.05	0.06
	As Min (mg/kg)	As Mean (mg/kg)	As max (mg/kg)	Cr Min (mg/kg)	Cr Mean (mg/kg)	Cr max (mg/kg)	Hg Min (mg/kg)	Hg Mean (mg/kg)	Hg max (mg/kg)
Legal limits		2.0 mg As/kg			-			0.1 mg Hg/kg	
	BLD	BLD	BLD	0.39	0.41	0.42	BLD	BLD	BLD

In the absence of clearly defined legal thresholds for heavy metals in corn-based concentrates intended for equine feed, the evaluation was conducted by reference to the maximum permissible levels established for forage materials (such as grass and hay), as outlined in Directive 2002/32/EC, Annex I. The following limits were considered: Lead (Pb) – 10.0 mg/kg, Cadmium (Cd) – 1.0 mg/kg, Mercury (Hg) – 0.1 mg/kg, Arsenic (As) – 2.0 mg/kg, and Fluorine (F) – 30.0 mg/kg (specific to complete or complementary feeds for adult horses). These values are expressed on a dry matter basis and were used as comparative benchmarks for assessing the safety of the concentrate samples collected in the control area.

Table S24. Legal Thresholds for Heavy Metals in Agricultural Soils (Topsoil 0–10 cm, Dry Weight Basis) According to Romanian Guidelines

Heavy Metal	M.P.L.* (mg/kg)	Alert Threshold (mg/kg) (susceptible / less susceptible)	Intervention Threshold (mg/kg) (susceptible / less susceptible)	Soil Susceptibility (definition)	Legal Source / Reference	Remarks
Copper (Cu)	20	100 / 250	200 / 500	Defined by soil buffering capacity (texture, organic matter)	Order 756/1997, Annex 1	Essential micronutrient; excess inhibits plant growth.
Zinc (Zn)	100	300 / 700	600 / 1500	Differentiated based on soil type	Order 756/1997, Annex 1	Mobile in acidic soils; high values toxic to crops.
Lead (Pb)	20	50 / 250	100 / 1000	Higher risk in sandy soils	Order 756/1997, Annex 1	Strongly accumulates in soil; persistent and neurotoxic.
Cadmium (Cd)	1.0	3 / 5	5 / 10	More mobile in acidic soils	Order 756/1997, Annex 1	Highly toxic; bioaccumulates in plants and food chain.
Nickel (Ni)	20	75 / 200	150 / 500	Toxicity depends on soil pH	Order 756/1997, Annex 1	At elevated levels inhibits root growth.
Cobalt (Co)	15	30 / 100	50 / 250	Stronger retention in clay soils	Order 756/1997, Annex 1	Essential for ruminants; excess may impair health.
Arsenic (As)	5.0	15 / 25	25 / 50	High persistence in mining zones	Order 756/1997, Annex 1	Potent toxicant; associated with mining/tailings.
Chromium (Cr)	30	100 / 300	300 / 600	Cr(VI) >> Cr(III) toxicity	Order 756/1997, Annex 1	Cr(VI) highly toxic and mobile; Cr(III) less bioavailable.
Mercury (Hg)	0.1	1 / 4	2 / 10	More mobile in organic soils	Order 756/1997, Annex 1	Persistent toxicant; severe effects at low levels.

Notes: All values are expressed as mg/kg dry weight (DW) for the 0–10 cm topsoil horizon. M.P.L. = Maximum Permissible Limit (background concentration not expected to pose risk). Alert thresholds denote concentrations above which soils require monitoring; intervention thresholds denote concentrations that demand risk management and/or remediation. "Susceptible soils" are sandy or weakly buffered soils with low organic matter; "less susceptible soils" are clay-rich or organic-rich soils with higher buffering capacity. Legal source: Order of the Ministry of Waters, Forests and Environmental Protection No. 756/3 November 1997 (Annex 1), approving the regulation on the assessment of environmental pollution, Bucharest, Romania.

Table S25. Assessment of Heavy Metal Accumulation in Soil Samples Across Three Zones Based on Legal Soil Quality Standards: A Descriptive Comparison

**Zone I**	**Cu Min (mg/kg)**	**Cu Mean (mg/ kg)**	**Cu max (mg/ kg)**	**Zn Min (mg/ kg)**	**Zn Mean (mg/ kg)**	**Zn max (mg/L)**	**Pb Min (mg/ kg)**	**Pb Mean (mg/ kg)**	**Pb max (mg/ kg)**
Legal limits		20 mg Cu/kg			100 mg Zn/kg			20 mg Pb/kg	
	56.68	74.70	95.37	288.06	357.41	438.13	25.13	30.33	36.16
	Cd Min (mg/ kg)	Cd Mean (mg/kg)	Cd max (mg/kg)	Ni Min (mg/kg)	Ni Mean (mg/kg)	Ni max (mg/kg)	Co Min (mg/kg)	Co Mean (mg/kg)	Co max (mg/kg)
Legal limits		1 mg Cu/kg			20 mg Pb/kg			15 mg Pb/kg	
	2.74	3.13	3.54	22.96	25.49	28.91	18.61	19.85	21.03
	As Min (mg/kg)	As Mean (mg/kg)	As max (mg/kg)	Cr Min (mg/kg)	Cr Mean (mg/kg)	Cr max (mg/kg)	Hg Min (mg/kg)	Hg Mean (mg/kg)	Hg max (mg/kg)
Legal limits		5.00 mg As/kg			30 mg Pb/kg			0.10 mg/Hg/kg	
	BLD	BLD	BLD	BLD	BLD	BLD	BLD	BLD	BLD
Zone II	Cu Min (mg/kg)	Cu Mean (mg/ kg)	Cu max (mg/ kg)	Zn Min (mg/ kg)	Zn Mean (mg/ kg)	Zn max (mg/L)	Pb Min (mg/ kg)	Pb Mean (mg/ kg)	Pb max (mg/ kg)
Legal limits		20 mg Cu/kg			100 mg Zn/kg			20 mg Pb/kg	
	70.52	156.71	277.17	191.08	416.86	709.31	13.59	30.68	42.26
	Cd Min (mg/ kg)	Cd Mean (mg/kg)	Cd max (mg/kg)	Ni Min (mg/kg)	Ni Mean (mg/kg)	Ni max (mg/kg)	Co Min (mg/kg)	Co Mean (mg/kg)	Co max (mg/kg)
Legal limits		1 mg Cu/kg			20 mg Pb/kg			15 mg Pb/kg	
	1.93	2.71	3.58	19.73	23.01	12.56	13.88	17.14	22.22
	As Min (mg/kg)	As Mean (mg/kg)	As max (mg/kg)	Cr Min (mg/kg)	Cr Mean (mg/kg)	Cr max (mg/kg)	Hg Min (mg/kg)	Hg Mean (mg/kg)	Hg max (mg/kg)
Legal limits		5.00 mg As/kg			30 mg Pb/kg			0.10 mg/Hg/kg	
	BLD	BLD	BLD	BLD	BLD	BLD	BLD	BLD	BLD
Zone III	Cu Min (mg/kg)	Cu Mean (mg/ kg)	Cu max (mg/ kg)	Zn Min (mg/ kg)	Zn Mean (mg/ kg)	Zn max (mg/L)	Pb Min (mg/ kg)	Pb Mean (mg/ kg)	Pb max (mg/ kg)
Legal limits		20 mg Cu/kg			100 mg Zn/kg			20 mg Pb/kg	
	8.95	9.20	9.45	42.05	50.65	65.12	12.50	12.87	13.26
	Cd Min (mg/ kg)	Cd Mean (mg/kg)	Cd max (mg/kg)	Ni Min (mg/kg)	Ni Mean (mg/kg)	Ni max (mg/kg)	Co Min (mg/kg)	Co Mean (mg/kg)	Co max (mg/kg)
Legal limits		1 mg Cu/kg			20 mg Pb/kg			15 mg Pb/kg	
	0.41	0.57	0.74	11.84	12.32	12.56	8.95	9.01	9.08

	As Min (mg/kg)	As Mean (mg/kg)	As max (mg/kg)	Cr Min (mg/kg)	Cr Mean (mg/kg)	Cr max (mg/kg)	Hg Min (mg/kg)	Hg Mean (mg/kg)	Hg max (mg/kg)
Legal limits		5.00 mg As/kg			30 mg Pb/kg			0.10 mg/Hg/kg	
	BLD	BLD	BLD	BLD	BLD	BLD	BLD	BLD	BLD
Soil samples exposed to anthropogenic sources of heavy metals pollution									
Huzum et al. (2012) (mg/kg) [59]	256.00	60.10	12.90	0.21	29.90	7.20	11.20	208.00	–
Bora et al. (2020) (mg/kg) [60]	621.79–4155.95	45.36–3483.25	6.62–4262.23	0.12–32.53	6.97–28.60	5.08–29.57	1.15–5.13	2.72±0.65	0.034–0.070
Chakraborty et al. (2017) (mg/kg) [61]	19.8–2760.0	54.4–2370.0	38.0–14,329.0	–	–	–	7.8–889.0	–	–
Paulette et al. (2015) (mg/kg) [62]	77–7675	–	705–10,074	–	–	–	–	–	–
Mihali et al. (2017) (mg/kg) [63]	40.9–621.6	82.26–1002	48.12–3472	0.04–11	4.98–9.06	3.3–8.2	0.61–80.1	–	–
Albulescu et al. (2009) (mg/kg) [64]	36.63–112.00	–	21.90	1.77	24.55	–	–	13.32	–
Bora et al. (2023) (mg/kg) [42]	3286.65–0.78	2834.58–12.56	1205.57–0.02	6.33–0.03	7.99–0.57	5.98–0.25	4.09–0.13	10.75–0.13	–
Bora et al. (2015) (mg/kg) [65]	479.64 ± 53.97	69.44 ± 4.02	14.77 ± 0.74	0.45 ± 0.10	16.28 ± 2.01	9.75 ± 1.47	–	–	–
Background soil samples									
European Communities Council 1986 (mg/kg) [66]	50–140	150–300	50–300	1–3	30–75	–	–	–	1–1.5
Kabata-Pendias, 2001 (mg/kg) [66]	13–24	45–100	22–44	0.37–0.78	12.0–34	–	0–9.3	–	–
Common abundance in topsoil (mg/kg) [66]	5–50	10–100	–	0.1–1	20–50	–	0.1–55	–	–
Phytotoxic levels of elements in soils (mg/kg) [66]	36–698	100–1.000	–	–	100	–	200	–	–
Fechete et al. (2024) (mg/kg) [67]	0.89 ± 0.33	24.66 ± 6.40	3.75 ± 2.98	BLD	2.27 ± 0.96	BLD	BLD	3.13 ± 0.93	BLD

Note: Min = minimum; Mean = arithmetic mean; Max = maximum; BLD = below detection limit. All concentrations are expressed on a dry weight basis (mg/kg). The legal limits presented correspond to the maximum admissible concentrations for contaminants in feed and food, as established by the European Union regulatory framework (e.g., Regulation (EC) No 1881/2006 setting maximum levels for certain contaminants in foodstuffs; Directive 2002/32/EC on undesirable substances in animal feed, and subsequent amendments). These thresholds serve as reference benchmarks for evaluating the safety of the investigated samples in relation to potential risks for animal and human health. BLD = Below the detection limit (LoQ): LoQ for Pb: 0.231 μg/L, LoQ for Cd: 0.069 μg/L, LoQ for Co: 0.136 μg/L, LoQ for As: 0.743 μg/L; LoQ for Hg 0.1379 μg/L.

Table S26. Summary of environmental and biological matrices for heavy metal assessment in horses

Matrix	Dominant Metals	Biomonitoring Value
Water	Cu, Zn, Pb, Ni, Co (Cd, As, Hg = BLD)	Primary exposure source; frequent exceedance of legal drinking-water limits.
Soil	Cu, Zn, Pb, Cd, Ni, Co (As, Hg = BLD)	Major reservoir of contamination; values often above soil quality standards.
Forage (Grass/Hay)	Pb, Cd, Cu, Zn, Ni (As, Hg = BLD)	Direct transfer route into the food chain; frequent exceedance of feed safety limits.
Concentrates (Control)	Low levels; all within legal limits	Safe baseline feed; confirms Zone III as uncontaminated reference.
Hair	As, Cu, Cr, Pb, Zn, Ni, Cd (Co variable, Hg BLD)	Chronic biomarker; captures long-term exposure, consistent across mane/tail.
Hoof	Cu, Zn, Pb, Ni, Co (Cr, As, Hg = BLD)	Chronic biomarker; wall accumulates more than sole, but both reflect exposure.
Serum	Ni, Co (Cu, Zn variable; others BLD)	Acute/short-term marker; Ni and Co are reliable discriminators of pollution impact.
Synovial fluid	Cu, Zn (others BLD)	Weak biomarker; high variability, limited diagnostic value.

Note: This table highlights how environmental reservoirs of heavy metals (water, soil, and forage) directly translate into biological burdens in horses. Hair and hoof emerge as the most powerful long-term sentinels of exposure, while serum captures acute changes and synovial fluid contributes little diagnostic value. The combined view emphasizes the necessity of monitoring across multiple matrices to fully understand both contamination pathways and their biological consequences.

Table S27. Percentage increase of dominant heavy metals in horses and environmental matrices (polluted vs. control zones)

Matrix	Metals with Increase	Approximate % Increase vs. Control
Water	Cu, Zn, Pb, Ni, Co	Cu +4950–+43,900%; Zn +270–+2130%; Pb +70–+1700%; Ni +290–+790%; Co +1560–+11,900%
Soil	Cu, Zn, Pb, Cd, Ni, Co	Cu +520–+2900%; Zn +190–+1280%; Pb +10–+230%; Cd +160–+770%; Ni +60–+140%; Co +55–+150%
Forage (Grass/Hay)	Pb, Cd, Cu, Zn, Ni	Pb +40–+1350%; Cd +130–+1520%; Cu +2500–+9100%; Zn +230–+1230%; Ni newly detectable
Concentrates (Control)	All metals low	Within legal feed limits; no significant increases
Hair	As, Cu, Cr, Pb, Zn, Ni, Cd	As +580–+3150%; Cu +1000–+1800%; Cr +330–+740%; Pb +180–+470%; Zn +170–+285%; Ni +125–+312%; Cd +110–+300%
Hoof	Cu, Zn, Pb, Ni, Co	Cu +95–+210%; Zn +75–+155%; Pb +300–+600%; Ni +110–+250%; Co +200–+425%
Serum	Ni, Co (Cu, Zn variable)	Ni +720–+980%; Co newly detectable; Cu +100–+280% (NS); Zn +10–+34% (NS)
Synovial fluid	Cu, Zn	Cu variable (−40% to +80%); Zn +110–+210% (high variability, NS)

Note: The percentage increases reported across matrices clearly illustrate the magnitude of heavy metal amplification in polluted zones compared with controls. Water and soil show extreme enrichments, with Cu, Zn, Pb, Ni, and Co rising by several orders of magnitude, while forage demonstrates a direct dietary pathway with dramatic increases in Pb, Cd, and Cu. Among biological

matrices, hair captures the broadest spectrum of elements with strong amplifications, confirming its role as the most reliable chronic biomarker, followed by hoof, which reflects long-term accumulation patterns. Serum provides valuable evidence of acute exposure, particularly for Ni and Co, whereas synovial fluid shows high variability and little diagnostic utility. Taken together, these findings highlight how environmental contamination is strongly mirrored in biological tissues, reinforcing the hierarchical value of hair and hoof for chronic monitoring, and serum for short-term exposure assessment.

Table S28. Environmental sources, dominant metals, EU legal compliance, and biomarker relevance in horses

Matrix	Dominant Metals	EU Legal Compliance	Biomarker Relevance
Water	Cu, Zn, Pb, Ni, Co (Cd, As, Hg = BLD)	Exceedances for Cu, Zn, Pb vs. EU drinking water limits	Primary exposure source
Soil	Cu, Zn, Pb, Cd, Ni, Co (As, Hg = BLD)	Exceedances for Cu, Zn, Pb, Cd vs. EU soil quality standards	Reservoir of contamination
Forage (Grass/Hay)	Pb, Cd, Cu, Zn, Ni (As, Hg = BLD)	Pb and Cd often at/above EU feed safety limits; Cu and Zn enriched	Direct transfer into food chain
Concentrates (Control)	All metals within legal limits	Fully compliant with EU feed standards	Safe baseline; reference matrix
Hair	As, Cu, Cr, Pb, Zn, Ni, Cd (Co variable)	Not directly regulated; reflects exposure to metals above legal thresholds	Best chronic biomarker (multi-metal, robust)
Hoof	Cu, Zn, Pb, Ni, Co (Cr, As, Hg = BLD)	Not regulated; shows accumulation linked to exceedances in soil/forage	Strong chronic biomarker (wall > sole)
Serum	Ni, Co (Cu, Zn variable; others BLD)	Not regulated; reflects short-term exceedances in exposure	Acute biomarker (Ni/Co most reliable)
Synovial fluid	Cu, Zn (others BLD)	Not regulated; poor sensitivity to environmental exceedances	Weak biomarker; limited diagnostic utility

Note: Water, soil, and forage from polluted zones show clear exceedances of EU legal thresholds for Cu, Zn, Pb, and Cd, confirming their role as the primary reservoirs and vectors of heavy metal exposure, while corn-based concentrates from the control area remain fully compliant, serving as a safe reference baseline. Among biological matrices, hair and hoof stand out as robust chronic biomarkers that closely mirror these environmental exceedances, whereas serum provides insight into short-term exposure peaks (especially for Ni and Co), and synovial fluid demonstrates poor sensitivity. The convergence of environmental and biological evidence underscores the sentinel value of horses for ecosystem monitoring and highlights the urgent need for systematic regulatory control in mining-impacted areas.
